# Stability of Bimetallic Pt_*x*_Ru_*y*_ – From Model Surfaces to Nanoparticulate Electrocatalysts

**DOI:** 10.1021/acsmaterialsau.3c00092

**Published:** 2024-01-16

**Authors:** Attila Kormanyos, Pascal Büttner, Michael Bosch, Maria Minichova, Andreas Körner, Ken J. Jenewein, Andreas Hutzler, Karl J. J. Mayrhofer, Julien Bachmann, Serhiy Cherevko

**Affiliations:** † Helmholtz Institute Erlangen-Nürnberg for Renewable Energy (IEK-11), Forschungszentrum Jülich, Cauerstr. 1, 91058 Erlangen, Germany; ‡ Department of Physical Chemistry and Materials Science, University of Szeged, Aradi sq. 1, Szeged 6720, Hungary; § Chemistry of Thin Film Materials, IZNF, Friedrich-Alexander-Universität Erlangen-Nürnberg (FAU), Cauerstr. 3, 91058 Erlangen, Germany; ∥ Department of Chemical and Biological Engineering, Friedrich-Alexander-Universität Erlangen-Nürnberg, Egerlandstr. 3, 91058 Erlangen, Germany

**Keywords:** bimetallic electrocatalyst, material library, dealloying, electrocatalyst stability, online ICP-MS

## Abstract

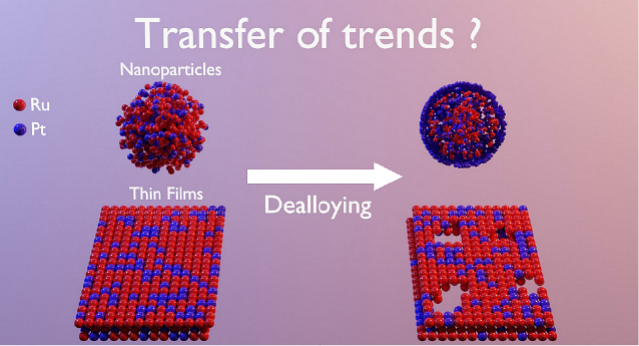

Fundamental research campaigns in electrocatalysis often involve the use of model systems, such as single crystals or magnetron-sputtered thin films (single metals or metal alloys). The downsides of these approaches are that oftentimes only a limited number of compositions are picked and tested (guided by chemical intuition) and that the validity of trends is not verified under operating conditions typically present in real devices. These together can lead to deficient conclusions, hampering the direct application of newly discovered systems in real devices. In this contribution, the stability of magnetron-sputtered bimetallic Pt_*x*_Ru_*y*_ thin film electrocatalysts (0 at. % to 100 at. % Ru content) along with three commercially available carbon-supported counterparts (50–67 at. % Ru content) was mapped under electrocatalytic conditions in acidic electrolytes using online ICP-MS. We found several differences between the two systems in the amount of metals dissolved along with the development of the morphology and composition. While the Pt-rich Pt_*x*_Ru_*y*_ compositions remained unchanged, 30–50 nm diameter surface pits were detected in the case of the Ru-rich sputtered thin films. Contrastingly, the surface of the carbon-supported NPs enriched in Pt accompanied by the leaching of a significant amount of Ru from the alloy structure was observed. Change in morphology was accompanied by a mass loss reaching around 1–2 wt % in the case of the sputtered samples and almost 10 wt % for the NPs. Since Pt_*x*_Ru_*y*_ has prime importance in driving alcohol oxidation reactions, the stability of all investigated alloys was screened in the presence of isopropanol. While Pt dissolution was marginally affected by the presence of isopropanol, several times higher Ru dissolution was detected, especially in the case of the Ru-rich compositions. Our results underline that trends in terms of electrocatalytic activity and stability cannot always be transferred from model samples to systems that are closer to the ones applied in real devices.

## 1 Introduction

Alcohols provide a promising alternative to H_2_ as an energy carrier. One of the main advantages is their physical state: liquid fuels can be easily stored, handled, and distributed via the already existing infrastructure established for gasoline.^1^ Moreover, their energy density is in the ballpark of gasoline (6–10 kW h kg^–1^).^2^ Beyond fuel cell applications, the electrocatalytic oxidation of various alcohols can potentially replace the oxygen evolution reaction (OER), which is commercially applied as the anode process in CO_2_ electrolyzers. By selecting the appropriate substrate molecule (considering the overall carbon-neutral/negative operation of the electrolyzer cell from CO_2_ and alcohol source to the final products), this could increase the energy efficiency of CO_2_ electrolyzers in parallel, providing valuable precursors for fine chemicals and fuels for the transportation industry. Besides alcohol oxidation, their electrocatalytic reduction could also play an important role in the future as a cathode process again, yielding precursor molecules with high market value.^3^ In contrast to the OER, electrocatalytic alcohol oxidation can be efficiently driven at lower overpotentials. By carefully selecting the alcohol and using the appropriate electrocatalyst, products in the anode compartment can be precisely tailored. As a result, electrolyzer operation is possible at lower cell voltages (higher energy efficiency), and value-added products are formed in both cell compartments (cost-effectiveness).^4−6^

The most simple case is when a pristine metal is used as the electrocatalyst. Platinum is an evident choice for the electrocatalytic oxidation of alcohols proceeding at low overpotentials and relatively high current densities, which is demonstrated extensively in the literature.^5,7,8^ However, Pt suffers from a set of disadvantages in alcohol oxidation reactions. For example, the alcohol oxidation proceeds at the highest achievable rate (around +0.70 V_RHE_ for methanol oxidation)^7^ at potentials where its surface starts to oxidize;^9^ the reaction stops whenever the Pt surface is covered with a compact layer of PtO_*x*_ species.^10^ Additionally, intermediates formed during the reaction (exclusively CO in the case of methanol oxidation)^7,11^ can irreversibly adsorb at the catalyst surface, poisoning it.^12,13^ Both scenarios first lead to a decrease in the achievable current density and subsequently to the complete deactivation of the catalyst. One strategy that was introduced to circumvent this issue is to use bimetallic alloys instead of the pristine noble metal. PtRu shows high activity toward the electrocatalytic oxidation of numerous alcohols, in fact, it is the state-of-the-art electrocatalyst for electrocatalytic oxidation of primary alcohols.^14^ Its first application for electrocatalytic methanol oxidation was demonstrated in the 1960s,^15^ and its popularity has been unbroken since.^16^ The high activity and its resistance to surface poisoning are rooted in the bifunctional alcohol oxidation mechanism, which was decoded three decades ago.^7,11^ In short, the role of Pt is the dehydrogenation of the given alcohol, forming carbonaceous intermediates, while oxygen-containing species nucleate at the surface of Ru atoms (several hundred mVs less positive than in the case of Pt). At optimal catalyst structure and composition, where an ensemble of Pt and Ru atoms is adjacent, the complete oxidation of the given alcohol is facilitated.

Besides activity and selectivity, the stability of the catalyst used under electrocatalytic conditions is the third descriptor that determines whether the given system can be used in applications. The stability of single metals from a thermodynamic standpoint can be described with Pourbaix diagrams.^17^ The combination of thermodynamic data with the *E* vs pH diagrams allows for the identification of the thermodynamically stable species at the given pH and potential. However, Pourbaix diagrams provide no information on kinetics, which is a serious deficiency considering that electrocatalytic reactions always proceed far from equilibrium, ideally at high current densities. Our group has invested significant efforts to link intrinsic metal properties (strength of M–M and M–O bonds) to the stability of *single metals* defining stability descriptors that can be applied to d-metals.^18^ Under oxidation of the metal electrocatalyst surface, oxygen atoms are incorporated into the crystal lattice, which leads to the breaking of M–M bonds. If the energy demand of this process is small, then the dissolution tendency increases through the formation and dissolution of the undercoordinated metal sites. Under reduction, the amount of oxide will determine the rate of its dissolution (depending on both the M–M and M–O bond strengths) and reduction. While we have been able to experimentally establish this correlation for pure single metals, at this point, it is impossible to do so even for binary systems. In our opinion, the field desperately needs more theoretical and experimental research efforts to understand the degradation mechanism of such complex systems. In terms of experiments, the whole composition space has to be mapped^19^ to make valid conclusions, which is hard and often impossible for samples typically employed in real devices (nanoparticles/supported nanoparticles – NPs).^20,21^ These can be substituted with model systems synthesized with an appropriate structure and composition.^19^ Material libraries are a perfect example of such model systems, allowing the simultaneous synthesis of the whole composition space ideally in a single step. Magnetron co-sputtering is an efficient tool for this purpose, allowing for the uniform and highly reproducible synthesis of the entire library.^22^ Moreover, since all samples are deposited on a single wafer, further characterization (physical, electrochemical, etc.) can be carried out in a high-throughput manner.^22,23^ While these samples are ideal model systems that can provide key fundamental insights, transferring the discovered trends in the activity and stability to electrocatalysts employed in real devices is not straightforward at all.

Dealloying (both from compositional and morphological aspects) of bimetallic non-noble/noble metal alloys employed for the oxygen reduction reaction (ORR) is an extensively studied phenomenon.^24−30^ In the case of these materials, a “pre-catalyst” is synthesized, and the active form of the catalyst is developed through an activation period during which the less stable component of the alloy is gradually dissolved. The superior activity is speculated to be achieved because of three reasons: (1) alteration of the band structure by the electronic/ligand effect (binding between the electrocatalyst surface and adsorbates), (2) strain in the crystal lattice caused by the atomic arrangement of surface atoms to reduce the lattice mismatch (geometric effect), and (3) as a result of dealloying, small groups of different metal atoms at the electrocatalyst surface act as preferential active sites.^31^ It has also been discovered that the dealloying process results in different morphologies, depending on the size of the NPs. Well-known examples of this category are PtNi,^24−26^ PtCo,^27^ and PtCu^27−30,32^ used as electrocatalysts in the ORR or IrNi^33^ and IrCo^34^ utilized as OER catalysts. On the other hand, there are considerably fewer reports in the literature for bimetallic systems, in which *both alloy constituents* are *noble metals*. Such alloys are, for example, PtRh,^35,36^ PdAu,^31,37^ and PtRu.^38^ There are even fewer studies exploring the whole composition space of the given bimetallic system, making it impossible to fully understand the mechanism of the dissolution process.

In this study, the stability of bimetallic Pt_*x*_Ru_*y*_ alloys was investigated. A scanning flow cell coupled to an inductively coupled plasma mass spectrometer (online ICP-MS) was utilized for this purpose, allowing for the quantification of the metal ions dissolved during the electrochemical protocol in real-time with high precision.^21,39,40^ First, the stability of a magnetron co-sputtered material library was screened in acidic media covering the entire alloy composition space (0–100 at. % Pt content, 10 at. % increments). Electrochemical protocols were designed to cover a wide potential window and somewhat mimic those typically performed during accelerated stress tests. We found that the stability of the alloy was significantly influenced by the Ru content, just like Ni, Co, and Cu in the case of PtM systems. The transfer of the observed trends was validated by carrying out an identical electrochemical protocol as in the case of the sputtered thin films but using commercially available carbon-supported Pt_*x*_Ru_*y*_ NPs. Besides composition, the evolution of the morphology was also monitored. We found that while NPs favored the formation of a dealloyed core–shell structure, 30–50 nm cavities formed in the case of the sputtered thin films. Our results point out that one must be careful before transferring activity and stability trends from model systems to samples that are potentially applied in real devices.

## 2 Experimental Section

### 2.1 Chemicals and Materials

HClO_4_ (70%, Suprapur), and isopropanol (C_3_H_7_OH, abbreviated as “IPA”, ACS, 99.5+%, VWR) were purchased from Merck and used without further purification. The carbon-supported Pt_*x*_Ru_*y*_ nanoparticles (Pt:Ru ratios 1:1, 1:1.5, and 1:2) were purchased from Tanaka. Ar (99.999%) was purchased from Air Liquide Deutschland GmbH. Sputter targets were purchased from HMW Hauner (Ru 99.9% and Au 99.99%) and Stanford Materials (Ti 99.7% and Pt 99.99%). All solutions in this study were prepared using ultrapure water (18.2 MΩ, TOC < 3 ppb) supplied by a Merck Millipore Milli-Q system.

### 2.2 Synthesis of Pt–Ru Thin-Film Material Libraries and Preparation of Carbon-Supported Nanoparticle Thin Films

For the Pt–Ru thin films, first native (100) silicon wafers (Silicon Materials Inc.) were cleaned by sonication in detergent (Hellamnex III, 2%), acetone, isopropanol, and DI water for 5 min each. Thereafter, they were treated with piranha solution (H_2_SO_4_/H_2_O_2_ 3:1) for 10 min right before further use. The metal films were grown using magnetron sputtering (CRC 622 model, Torr International, Inc.) with a base pressure, working pressure, and Ar flow of 1 × 10^–4^ Pa, 0.3 Pa, and 5 mL min^–1^, respectively. First, a thin adhesion layer of Ti/Au was grown with a gun-to-sample distance of 20 cm, active substrate rotation, and without breaking a vacuum in between Ti and Au depositions. For the gradient films, substrate rotation was turned off, and the substrate holder was moved closer to the sputter guns (gun-to-sample distance ∼10 cm and gun-to-gun distance ∼25 cm) to enhance the gradient deposition. Both sputter guns were run simultaneously to achieve a homogeneously mixed Pt/Ru gradient film of varying compositions along the sample length. Pure Pt and Ru films were grown using the same setup with only one of the guns operating. All experimental parameters are presented in Table 1.

**Table 1:** Sputter Parameters and Average Thickness of the Deposited Thin Films^a^

material	Ti	Au	Pt	Ru
RF/DC	RF	DC	DC	RF
power density [W cm^–2^]	2.55	1.28	1.28	2.55
deposition rate [Å s^–1^]	0.1	1.0	3.3	3.3
thickness [nm]	3	60	80	100

a RF = radio frequency, and DC = direct current.

The carbon-supported Pt_*x*_Ru_*y*_ nanoparticles were drop-casted on a glassy carbon (GC) substrate (SIGRADUR G, HTW 5 cm × 5 cm) to yield thin films. Before drop-casting, the GC substrate was ground and polished. An Md-Mol (cat. number 40500079) polishing pad together with a DiaPro Md-Mol paste (*d* = 3 μm, Struers) was used for polishing (150 N, 200 rpm speed for the polishing head, while the sample holder was counter-rotated at 150 rpm for a total of 5 min). The polished substrates were cleaned by sonicating them in Milli-Q water and isopropanol for 8 min each. Finally, each substrate was dried with KimWipes and stored under ambient conditions overnight before drop-casting of the catalyst inks. Inks were prepared by dispersing 3.3 mg of carbon-supported catalyst in 1 cm^3^ Milli-Q water. This was followed by sonication for 40 min at 25% intensity using a sonication horn (Branson SFX 150): after each 4 s of sonication, a 2 s break was introduced. The dispersion was cooled by an ice bath during sonication. 0.2 μL aliquots were drop-casted on the GC substrate resulting in approximately 20 μg cm^–2^ metal loading. All spots were dried under ambient conditions. Spot diameters were measured by a laser scanning microscope (Keyence VK-X250). All electrochemical and online dissolution data presented in this study were normalized to this individual geometric surface area.

### 2.3 Structural and Morphological Characterization

#### 2.3.1 X-Ray Photoelectron Spectroscopy (XPS) and X-Ray Diffractometry (XRD)

The surface composition of the sputtered samples was determined with XPS. XPS measurements were carried out on a PHI Quantera II scanning XPS microprobe (Physical Electronics, ULVAC-PHI). Samples were fixed on the sample holder using double-sided copper tape (the same tape was used to establish a contact between the wafer and the sample holder). Spectra were recorded by using Al Kα irradiation. A 200 μm diameter area was irradiated at 50 W and 15 kV. Survey scans were collected at 280 eV pass energy with a step size of 0.5 eV. All gathered data was further analyzed by CasaXPS (V2.3.18) using instrument-specific relative sensitivity factors. Shirley backgrounds and the binding energy scale were calibrated to the adventitious carbon peak at 284.8 eV.

X-ray diffractograms were measured on a Bruker D8 Advance instrument with a Cu Kα source and a LynxEye XE-T detector.

#### 2.3.2 Scanning Electron Microscopy and High-Angle Annular Dark Field Scanning Transmission Electron Microscopy and Energy Dispersive X-Ray Spectroscopy (SEM and HAADF-(S)TEM-EDXS)

Scanning electron microscopy micrographs were recorded on a JEOL JSM 6400 equipped with a LaB_6_ cathode and a SAMx energy-dispersive X-ray detector at an acceleration voltage of 20 kV or a Gemini 500 field-emission SEM from Carl Zeiss at an acceleration voltage of 2 kV.

High-angle annular dark field scanning transmission electron microscopy and energy dispersive X-ray spectroscopy was carried out using a Talos F200i (Thermo Fisher Scientific) equipped with two Bruker XFlash 6 | 100 EDS detectors. The microscope was operated at a primary electron energy of 200 keV. TEM samples were prepared from an ink described in Section 2.2. Prior to TEM measurements, Pt_*x*_Ru_*y*_/C samples drop-casted on the GC (either before or after the applied electrochemical protocol) were scratched by adding one droplet (20 μL of IPA) on each catalyst spot of interest. As a next step, the catalyst spot was scratched off with a polypropylene spatula and directly transferred to a Ni grid with an automatic pipette.

### 2.4 Electrochemical Measurements

All electrochemical measurements were gathered in a 0.1 M HClO_4_ solution. In some cases, 0.05 M isopropanol was also added to the electrolyte solution. The reason behind this relatively low concentration lies in the factory limitations of the ICP-MS (organics/salt content should not exceed 2 wt %). The protocols were carried out in a custom-designed scanning flow cell (SFC) built in-house.^39^ In all cases, the sputtered thin films or drop-cast NPs served as the working electrode, while a GC rod (SIGRADUR) was employed as the counter electrode. The counter electrode was connected to the SFC on the inlet side. A double-junction Ag/AgCl/3 M KCl electrode was used as the reference that was connected to the outlet of the SFC cell via a T-connector (preventing the contamination of the electrolyte solution with Cl^–^ anions). All electrolyte solutions were prepared freshly, and their pH was measured at the beginning of each measurement day. Potentials were converted to the reversible hydrogen electrode scale using these values. All electrochemical protocols were performed by using a Gamry Instruments (Reference 600) potentiostat. The flow of the electrolyte solution was regulated by a peristaltic pump (Ismatec). The working electrode was placed on an XYZ translational stage (Physik Instrumente, M-403). This allowed for precise positioning to screen the samples of interest rapidly. All instruments (mass flow controllers, potentiostat, gas control box, magnet valves, peristaltic pump, etc.) were controlled using an in-house-developed LabView software. Contact with the working electrode was established close to open circuit potential (OCP), +0.05 V_RHE_. The length of this hold was 5 min. It was followed by a potentiodynamic protocol recording three cyclic voltammograms (CVs) starting at +0.05 V_RHE_ by gradually increasing upper potential limits (UPL) of +0.90 V_RHE_, +1.20 V_RHE_, and +1.50 V_RHE_ applying a 10 mV s^–1^ scan rate. As a next step, the potential was held at +0.05 V_RHE_ for 2 min. Thirty CVs were recorded between +0.05 V_RHE_ and +1.50 V_RHE_ applying 200 mV s^–1^ scan rate in a subsequent step that was again followed by a 2 min potentostatic hold at +0.05 V_RHE_. The protocol was finished by gathering an additional CV between +0.05 V_RHE_ and 1.5 V_RHE_, applying a 10 mV s^–1^ scan rate. The described electrochemical protocol was used for all measurements, investigating the stability and morphological or compositional changes in this work. In addition, the electrochemical behavior was scrutinized by CVs recorded between +0.05 V_RHE_ and +1.20 V_RHE_ applying a 50 mV s^–1^ scan rate. One cleaning cycle between +0.05 V_RHE_ and +1.50 V_RHE_ applying the 200 mV s^–1^ scan rate was applied prior to recording the voltammograms.

### 2.5 Online ICP-MS Measurements

Online stability measurements were performed by connecting the outlet of the SFC to the inlet of an inductively coupled plasma mass spectrometer (PerkinElmer Nexion 350X). The electrochemical setup, along with the measurement protocol, was identical to that described in detail in Section 2.4. The instrument was calibrated daily by a four-point calibration slope diluted from standard solutions (Merck Centripur Pt, Ru, Re, Rh) containing the metals of interest in a given concentration in 0.1 M HClO_4_. ^187^Re and ^103^Rh served as the internal standards. Internal standards were introduced to the nebulizer of the ICP-MS together with the sample flow via a Y-connector right before the peristaltic pump of the instrument. The purged electrolyte flow rate was controlled by the peristaltic pump of the ICP-MS (M2, Elemental Scientific) and calibrated daily. The average flow rate was 3.52 ± 0.05 μL s^–1^.

## 3 Results and Discussion

### 3.1 Synthesis and Physico-Chemical Characterization

In this study, two sets of samples were investigated: (a) the model Pt_*x*_Ru_*y*_ thin film material library as typically used in fundamental studies and (b) carbon-supported Pt_*x*_Ru_*y*_ nanoparticles suited for application in real devices. The material library was synthesized by magnetron co-sputtering.

**Figure 1:**
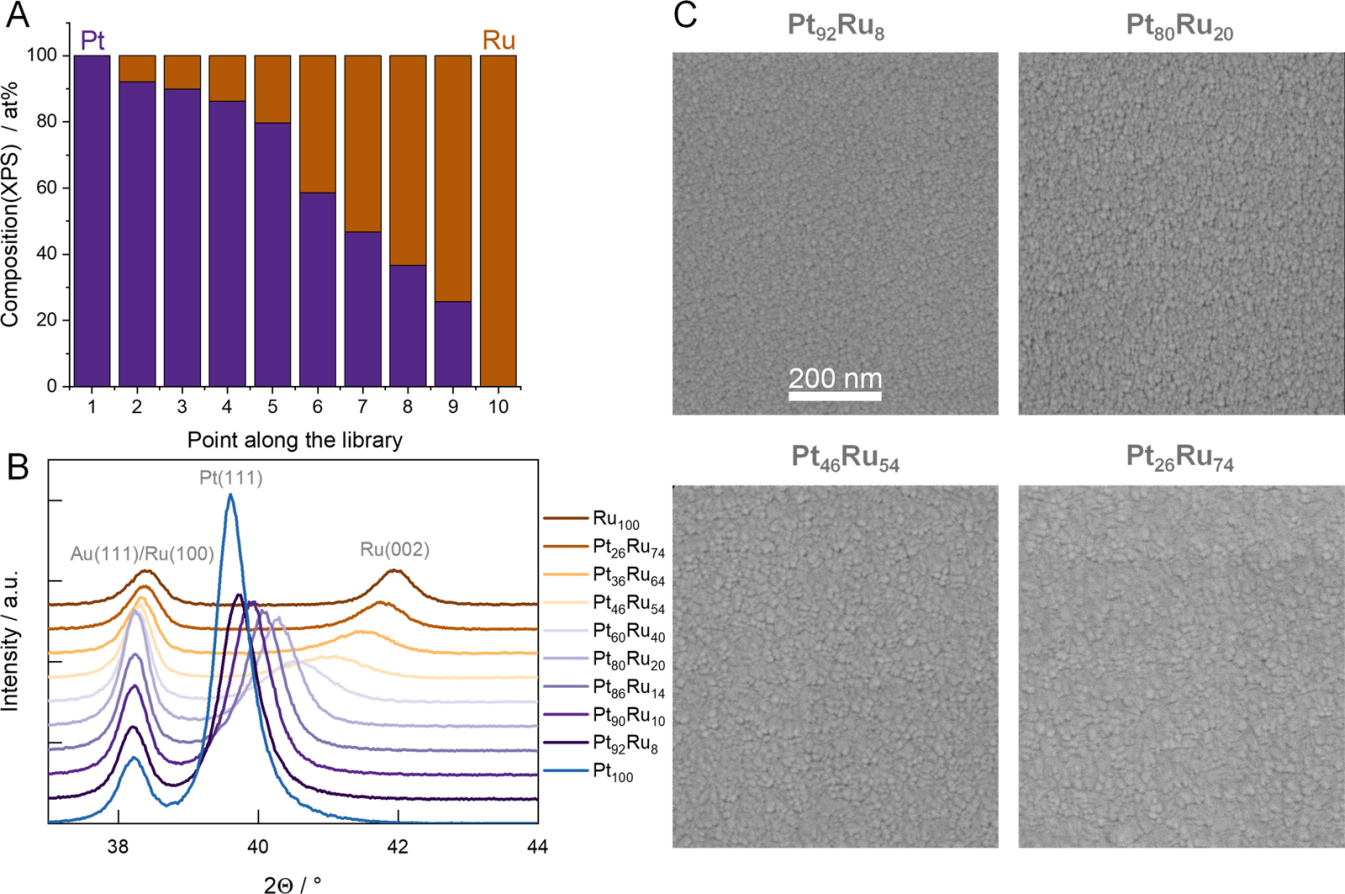
(A) Composition of sputtered Pt, Ru, and Pt_*x*_Ru_*y*_ thin films determined by XPS. (B) XRD data recorded for the sputtered Pt_*x*_Ru_*y*_ library. (C) Representative SEM images captured for Pt_*x*_Ru_*y*_ samples. The composition of each sample is written above the images and is determined by XPS.

Pristine Pt and Ru films were synthesized alongside the library for comparison. A total of 10 points were selected from the library, and 8 were used for the electrochemical and in situ stability measurements. The surface and bulk compositions of the samples were screened by X-ray photoelectron spectroscopy (XPS), energy-dispersive X-ray spectroscopy (EDX), and X-ray diffraction (XRD). It is visible in Figure 1A that the Pt_*x*_Ru_*y*_ ratio changed monotonically along the library, gradually shifting from a Pt-rich binary alloy to a Ru-rich system. The Pt (111) diffraction peak can be clearly identified for the Pt-rich sample centered at 39.6°.^41^ Interestingly, an additional diffraction peak was located at 38.2° corresponding to the Au adhesion layer.^42^ The Pt (111) reflection gradually shifts toward higher 2Θ values with the increasing amount of Ru and transitions to a Ru (002) reflection^41^ (centered at 42.96° in the case of the Ru-rich composition). A similar trend can be observed for the Au (111) reflection (coming from the adhesion layer), which first decreases in intensity and then shifts toward higher 2Θ values. This is because of the increasing amount of Ru in the PtRu lattice (the Ru (100) peak is located around 38.7° for pristine Ru). This leads to a size reduction of the unit cell in the Pt fcc lattice and then to a gradual transition to the Ru hcp lattice. There are two compositions among the ones scrutinizedin our study, namely, Pt_46_Ru_54_ and Pt_60_Ru_40_, in which case the single-phase nature of the samples is not evident. According to the literature,^43^ magnetron sputtered PtRu samples can exhibit two phases in this composition range (40–60 at. % Ru content). In the referenced example, however, both the Pt (111) and Ru (002) reflections are separated and clearly identifiable as opposed to our case where the two reflections appear to be merged and manifested as a single peak. All in all, we conclude that at least 8 out of the 10 sputtered thin films formed single-phase alloys (either fcc or hcp), while there are two compositions in which case the single-phase nature of the samples is questionable. These most likely formed a mixed fcc and hcp phase, in good agreement with the cited literature. The EDX data are presented in Figure S1. The morphology of the thin films was studied with SEM (see a couple of examples in Figure 1C, the rest is presented in Figure S2). Generally, the surface of all of the tested samples appears to be homogeneous. The only visible difference that can be spotted is the size of the crystallites: their diameter gradually increases with increasing Ru content. Similar trends were found for other sputtered bimetallic systems such as Pt_*x*_Ir_*y*_.^19,44^ However, in that case, the crystallite size decreased with the increasing amount of Ir.

Carbon-supported NPs were acquired from a commercial source and drop-casted on a GC plate after ink formulation. Composition of the samples was studied with XRD (Figure S3). Reflections characteristic of Pt (111) and Pt (200) are the only ones that can be discerned from the diffractogram recorded for the Pt_50_Ru_50_ sample, suggesting a single-phase alloy crystallized in an fcc lattice. When the amount of Ru is further increased, two notable changes occur: (i) reflections characteristic to hcp Ru (100) and (101) appear, and (ii) the Pt (111) and (200) become distorted. These asymmetries indicate a dual-phase microstructure for the two Ru-rich compositions, which are still predominantly fcc. Interestingly, the composition range in which PtRu forms two phases is notably different compared to the sputtered thin films (the C-supported Pt_50_Ru_50_ sample appears to be still a single phase, while the sputtered Pt_46_Ru_54_ sample appears to be phase separated). However, the conclusions for the C-supported NPs are similar to what the phase diagram of the bulk Pt–Ru alloys suggest (two phase region in between ≈60 and 80 at. % Ru content).^43^ The morphology of the carbon-supported NPs was scrutinized with a HAADF-STEM (Figure S4). Pt_*x*_Ru_*y*_ nanoparticles can be clearly spotted in the carbon matrix, with an even distribution in the case of all three samples. The average particle size is between 2 and 5 nm, which perfectly overlaps with the values provided by the manufacturer. The composition of the NPs was determined with EDX (Table S1); the Ru content ranges as follows: 49 at. %, 59 at. %, and 65.5 at. %.

### 3.2 Electrochemical Behavior

**Figure 2:**
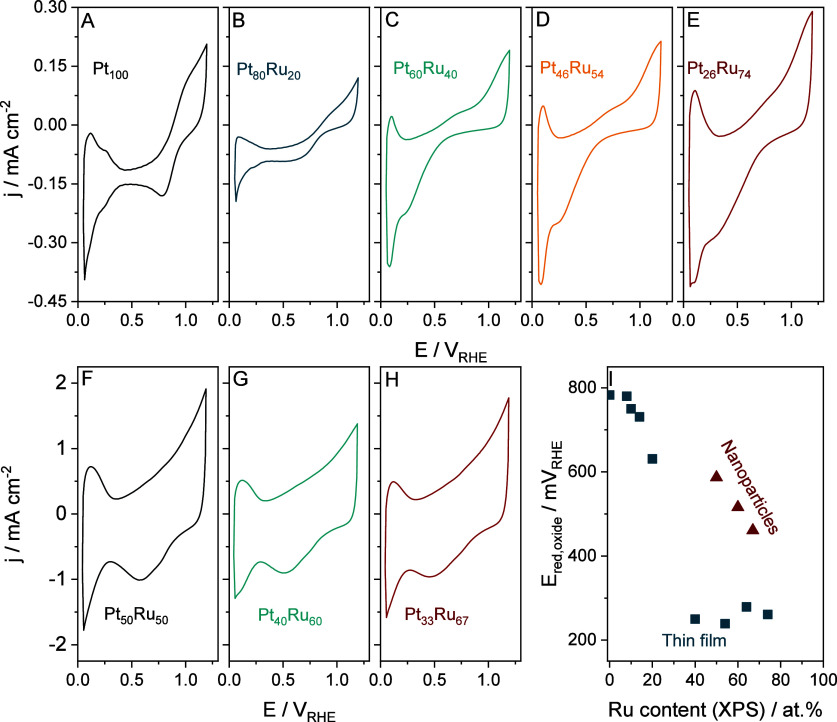
(A–E) Cyclic voltammograms recorded for the sputtered thin films and (F–H) C-supported nanoparticles. CVs were recorded between 0.05 V_RHE_ and 1.20 V_RHE_ by applying a 50 mV s^–1^ scan rate in Ar-saturated 0.1 M HClO_4_ electrolyte. Prior to recording the CVs presented here, a cleaning cycle was applied between 0.05 V_RHE_ and 1.50 V_RHE_ applying a 200 mV s^–1^ scan rate. (I) The position of the oxide reduction peak (derived from the CVs presented in A–H and in Figure S5) is a function of the Ru content. Teal squares: sputtered-thin films, and burgundy triangles: C-supported NPs.

The electrochemical behavior of both the thin films and carbon-supported NPs was screened by CV. CVs were recorded between 0.05 V_RHE_ and 1.20 V_RHE_ by applying a 50 mV s^–1^ scan rate. Measurements were performed in the SFC without connecting it to ICP-MS in Ar-saturated 0.1 M HClO_4_. The results are presented in Figure 2 (see also Figure S5 for the CVs recorded for the rest of the sputtered thin film library). Prior to collecting the CVs presented below, a rapid cleaning cycle was performed (between +0.05 V_RHE_ and +1.50 V_RHE_, 200 mV s^–1^ scan rate) to remove any native oxides/organic contaminants inherently present at the electrode surface stored under ambient conditions. The H_UPD_ region of the CVs is not symmetrical, with the whole voltammogram being shifted toward cathodic currents due to trace amounts of oxygen that could penetrate the SFC during the measurements despite the vigorous Ar saturation. Because of this, only qualitative observations can be made regarding the H_UPD_ region. The second notable difference between the voltammograms is the position of the oxide reduction peak. It is centered at +0.78 V_RHE_ for pristine Pt, corresponding to the reduction of the previously formed PtO_*x*_.^9,19^ The position of this peak is closely related to the amount of Pt present in the bimetallic alloy. There is a small but steady decrease until the Pt content reaches 80 at. %. If the alloy contains less Pt, the position of the oxide reduction peak shifts cathodically from +0.63 V_RHE_ to around +0.26 V_RHE_ and stabilizes at this value. No clear oxide reduction peak can be identified for pure Ru (Figure S5). Similar dependence was found for PtIr alloys.^19^

A similar set of CVs was recorded for the C-supported NPs. The cathodic shift of the H_UPD_ region can also be observed, but to a much smaller extent because the whole drop-casted spot fitted under the SFC opening (in contrast to the thin film material library), preventing the penetration of oxygen to the catalyst layer in an excess amount. The measured current densities are approximately one magnitude higher than in the case of the sputtered thin films, thanks to the higher electrochemically accessible surface area (ECSA). Additionally, while the area of the CVs differs notably in the case of the sputtered samples, the area of the CVs is similar for the NPs. This can be explained by the uniform size and loading of the NPs on the carbon matrix (as evidenced by the HAADF-STEM results), while crystallite sizes in the case of the sputtered samples show considerable differences (Figure 1C). The peaks on the reverse scan corresponding to the reduction of the previously formed surface oxide layer also shift to less positive potentials with increasing amounts of Ru. However, this shift seems to be more “even” than with the sputtered Pt_*x*_Ru_*y*_ samples. Here, *E*_red, oxide_ decreases from +0.59 V_RHE_ to +0.46 V_RHE_ when the amount of Ru increases from 50 at. % to 67 at. %, while this value was centered around ≈+0.25 V_RHE_ in the case of the sputtered samples with similar Ru contents.^45^ Cyclic voltammetry provides information about the topmost layer of the catalyst, and the position of the oxide reduction peak is in direct correlation with the amount of Pt in the top monolayer.^46^ Therefore, the observation presented above indicates that the sputtered Ru-rich samples contain more Ru at the electrode surface than one would expect from the bulk composition. More details on this phenomenon are described in the following sections.

### 3.3 Stability Under Electrocatalytic Conditions

The outlet of the SFC was connected to the inlet of an ICP-MS. Ar-saturated 0.1 M HClO_4_ was used as the electrolyte to screen the stability of both Pt and Ru in real time. As a first step, contact with the working electrode (i.e., not electrochemically precleaned sputtered thin films or carbon-supported NPs) was established close to the OCP, +0.05 V_RHE_ applied potential (for 5 min). It was followed by three CVs recorded between 0.05 V_RHE_ and gradually increasing upper potential limits (UPL) of +0.90 V_RHE_, +1.20 V_RHE_, and +1.50 V_RHE_ applying a 10 mV s^–1^ scan rate. To allow the ICP-MS signal to decrease to its baseline values, the potential was held at +0.05 V_RHE_ for 2 min. Thirty CVs were recorded between +0.05 V_RHE_ and +1.50 V_RHE_ applying a 200 mV s^–1^ scan rate in a subsequent step that was again followed by a 2 min potentiostatic hold at +0.05 V_RHE_. The protocol was finished by gathering an additional CV between +0.05 V_RHE_ and +1.5 0 V_RHE_ by applying a 10 mV s^–1^ scan rate. The protocol was designed in such a way to (1) identify the potential region in which Pt and Ru either are stable or start to dissolve (CVs with increasing potential limit), (2) mimic the effect of accelerated stress tests (ASTs) on dissolution widely applied by the community (30 fast cycles), and (3) determine how the stability is altered after the AST protocol (similar CV before and after the AST). The potential window is wider than that typically experienced by the anode electrocatalyst layer in a direct alcohol fuel cell (DAFC) or electrolyzer cell under process conditions. However, there are specific scenarios when such high positive potential values can develop, for example, during the start/stop of the cell or when the electrolyte suddenly depletes in the oxidizable substance. An other example is that PtRu is applied as an anode in a water electrolyzer, in which case, the anode potential can reach values above +1.00 V_RHE_. All in all, it is important to cover these scenarios as well, hence the potential window employed in our protocol.

**Figure 3:**
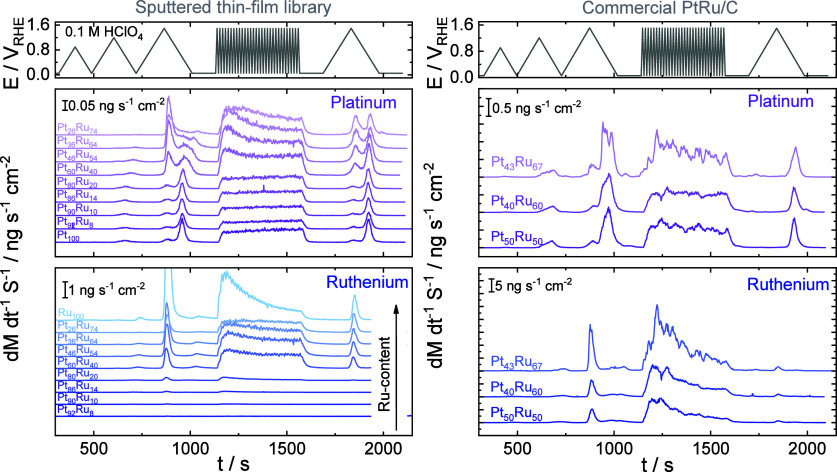
Dissolution rates of Pt and Ru for sputtered Pt_*x*_Ru_*y*_ samples (left panel) and for the C-supported NPs (right panel) recorded during an electrochemical protocol consisting of three CVs with increasing upper potential limit from +0.90 V_RHE_ to +1.50 V_RHE_ (Δ*E* = 0.30 V, υ = 10 mV s^–1^), and AST step where 30 CVs were recorded in between +0.05 V_RHE_ and +1.50 V_RHE_ applying a 200 mV s^–1^ scan rate. The protocol was finished with a CV recorded in between +0.05 V_RHE_ and +1.50 V_RHE_, applying a 10 mV s^–1^ scan rate. 0.1 M HClO_4_ saturated with Ar was used as an electrolyte for all measurements.

Results collected for the sputtered Pt_*x*_Ru_*y*_ thin films are presented on the left side of Figure 3. The three CVs recorded at 10 mV s^–1^ are discussed first. Magnified sections from both panels in Figure 3 are presented in Figure S6. No Pt dissolution was observed if the UPL was +0.90 V_RHE_, even for pure Pt. The onset of Pt dissolution was as high as +1.12 V_RHE_, which is in line with previous literature reports and reflects the sensitivity of the used ICP-MS setup.^9,47−49^ There are typically two dissolution features that can be identified for Pt in CV experiments: transient dissolution during the forward scan (often called “anodic dissolution,” and we follow this nomenclature in our study) originating from the formation of a PtO_*x*_ surface oxide layer first by place exchange and then by the formation of a continuous oxide passivation layer.^9,18^ The second transient dissolution peak is visible during the reverse scan (“cathodic dissolution”), corresponding to the reduction of the formed PtO_*x*_, likely PtO_2_,^50^ surface oxide layer. The magnitude of the cathodic dissolution feature is always higher for pristine Pt than for the anodic one. Interestingly, this behavior stops when the Ru content reaches 60 at. %: the anodic dissolution rate of Pt is consistently higher than the cathodic dissolution in the Ru-rich samples. This observation applies to the CVs collected up to +1.50 V_RHE_ UPL.

In contrast to Pt, Ru dissolution is visible (*E*_onset_ = +0.80 V_RHE_) during the first CV recorded to +0.90 V_RHE_ UPL. This value is determined by the limit of detection of the ICP-MS, which was 2.01 ×10^–3^ μg·dm^–3^ for Ru. The *E*_onset_ of dissolution is close to what one would expect from thermodynamics (+0.74 V_RHE_).^14,17^ However, the measured dissolution rates are so small in the case of the first cycle that it unequivocally can only be identified when the Ru content reaches 60 at. % in the alloy (see Figure S6 for the magnified sections of Figure 3). It is important to note here that this does not mean that no Ru dissolution occurs at lower Ru contents, only that Ru dissolution is below the detection limit of ICP-MS.

If the UPL is lower than +1.20 V_RHE_, then two dissolution features can be identified. A small anodic dissolution appears around +0.80 V_RHE_ due to the formation of Ru oxides (RuO_2_ · H_2_O, Ru(OH)_3_) first by place exchange, which is followed by the rapid passivation of the electrode surface. The anodic dissolution is followed by a cathodic dissolution corresponding to the reduction of the Ru oxides formed previously.^14,17,47,51^ If the UPL is increased further (+1.50 V_RHE_ in our case), the magnitude of anodic dissolution increases significantly. The dissolution of Ru above 1.40 V_RHE_ becomes constant instead of transient because of the formation of RuO_4_, which is a thermodynamically unstable intermediate (chemical/direct anodic dissolution).^51^

Looking at the dissolution curves on the left side of Figure 3, it seems that Pt dissolution depends on Ru dissolution in the Pt_*x*_Ru_*y*_ alloy. The ratio of anodic/cathodic dissolution peaks for Pt reversed when Ru dissolution became more pronounced. This is clearly visible when the UPL was set to +1.50 V_RHE_. The ratio between the anodic and cathodic peak dissolution rates is presented in Figure S7. A striking increase in the peak dissolution rates calculated for Pt can be observed when the amount of Ru reaches 40 at. %. This might be caused by the higher amount of Ru present at the alloy surface, as concluded from the shift in the metal oxide reduction peak position in Figure 2I. In contrast, anodic dissolution is always higher for Ru, and the anodic/cathodic peak ratio monotonously increases with Ru content. The reason behind the increase in Pt dissolution is because of Ru dissolution: (1) the extensive Ru dissolution dragged a lot of Pt from the crystal lattice and introduced them to the electrolyte, and (2) a lot of fresh Pt sites became available due to the gradual roughening of the electrode surface. This means that binary Pt_*x*_Ru_*y*_ alloys containing a high amount of Ru appear less stable than their Pt-rich counterparts.

Pt dissolution starts at +0.86 V_RHE_ and can be detected during the first CV in the case of the C-supported Pt_*x*_Ru_*y*_ samples, while Ru dissolution starts at +0.76 V_RHE_. As stressed above, the onset potential of dissolution strongly depends on the detection limit of the ICP-MS, which was 8.96 × 10^–3^ μg dm^–3^ and 2.01 × 10^–3^ μg dm^–3^ for Pt and Ru, respectively. The higher ECSA of the C-supported NPs allowed for the detection of both metals with higher precision. Thus, both values match well with the ones derived from the Pourbaix diagrams.^17^ It is visible that dissolution rates are approximately 1 order of magnitude higher than those measured for the sputtered thin films, thanks to the higher electrochemically active surface area of the nanoparticles. Additionally, the dissolution curves are considerably noisier. The most prominent difference between the data recorded for the sputtered samples and the C-supported NPs is the ratio of the anodic and cathodic dissolution peak for Pt (see Figure S7 for the ratios and their comparison to the ones obtained for the sputtered thin films). Here, the amount of Ru in the samples varied from 50 to 67 at. %. In contrast to the sputtered films, the cathodic dissolution was always higher than the anodic dissolution. It might originate from a similar phenomenon outlined in the case of the oxide reduction peak positions determined for the CVs (see Figure 2I), namely, it might be that the Ru-rich sputtered samples contain significantly more Ru at the close vicinity of the electrocatalyst surface while this seems not to be the case for the NPs. Apart from this, Pt dissolution rates are stagnant and do not scale with the amount of Pt in the alloy. Ru dissolution characteristics are identical to those observed for the sputtered thin films.

As a next step, a “mini-AST” protocol was performed, recording 30 cycles between +0.05 V_RHE_ and +1.50 V_RHE_ at a 200 mV s^–1^ scan rate. The purpose of this was to probe the stability of the alloy constituents over time. Due to the high scan rate, the transient dissolution of Pt reaches a stable value (≈ 0.065 ng s^–1^ cm^–2^) over the course of the experiment. It seems to be stable with a marginal increase until Pt is the major constituent of the alloy; dissolution rates increase notably when the amount of Pt in the alloy drops below 50 at. %. The shape of the whole dissolution feature changes from a straight line to a sudden increase, followed by a slow decay of the dissolution rate to a steady-state value. Unlike in the case of, for example, PtCu or AgAu bimetallic systems,^52−54^ no parting limit (a specific amount of the less noble constituent in the alloy below which no dealloying occurs, irrespective of the UPL applied) can be defined for PtRu. We speculate that this is because the nobility (i.e., redox potentials) of Pt and Ru does not differ significantly. Ru dissolution is already visible above 10 at. % Ru content, but it becomes dominant when the Ru content passes 50 at. % in the alloy. The shape of the dissolution feature is similar to that observed for Pt in the Ru-rich samples, implying that, as in the CVs case, Pt dissolution is heavily influenced by Ru dissolution when Ru is in excess. Ru dissolution is highest for the pristine Ru sample, while strikingly, the most Pt dissolves for the alloy containing the *LEAST* amount of Pt! Our observations are similar to what was published earlier for magnetron-sputtered Pt_*x*_Cu_*y*_ samples; Cu dissolution monotonously increased with the Cu content, while Pt dissolution rates did not change visibly (despite that considerably less Pt was present at the electrode surface).^55^ It has to be noted, however, that direct comparisons are of limited value since only a few compositions were characterized in these papers, in contrast to our study, where the whole composition range was screened. This picture is somewhat different for the C-supported NPs: the change in the shape of the dissolution feature for Pt occurs only at the highest Ru content (67 at. %), and the magnitude of Pt dissolution seems to not scale with the decreasing amount of Pt. The shape of the Ru dissolution curve is comparable to that recorded for the sputtered system.

Finally, the electrochemical protocol ended with recording one more CV between +0.05 V_RHE_ and +1.50 V_RHE_ by applying a 10 mV s^–1^ scan rate. The Pt dissolution features are identical to the ones recorded before the AST step for the sputtered, Pt-rich samples, while anodic dissolution decreases considerably for the Ru-rich thin films (becoming lower than or identical to the subsequent cathodic dissolution). The Ru dissolution measured is significantly lower than that before the AST step. This observation supports the theory outlined above that Pt dissolution is influenced by Ru dissolution. Here, it seems that the Ru-rich samples are somewhat depleted in Ru, preventing the excessive leaching of Pt from the crystal lattice. Only the anodic dissolution feature is visible for Ru and when the Ru-content exceeds 40 at. % (the one for Pt_60_Ru_40_ is barely visible). The magnitude of these dissolution rates is notably smaller than that recorded before the AST step. Only cathodic dissolution is clearly visible (anodic dissolution peaks are also present but almost blend in the baseline) for Pt in the case of the C-supported NPs, and again, dissolution rates are notably lower compared to the CV recorded before the AST step. This is even more striking for Ru: only a tiny anodic dissolution feature is visible, indicating that either Ru is in a form in which it cannot be dissolved from the alloy or the alloy is depleted in Ru.

In conclusion, several differences were identified in terms of dissolution characteristics between the sputtered thin-film material library and commercially acquired C-supported PtRu NPs. To make these qualitative observations quantitative, all of the dissolution features were integrated, yielding dissolved amounts. Based on these, the difference between the two investigated systems is scrutinized and discussed further in Section 3.4.

### 3.4 Amounts of Dissolved Pt and Ru under Electrocatalytic Conditions

**Figure 4:**
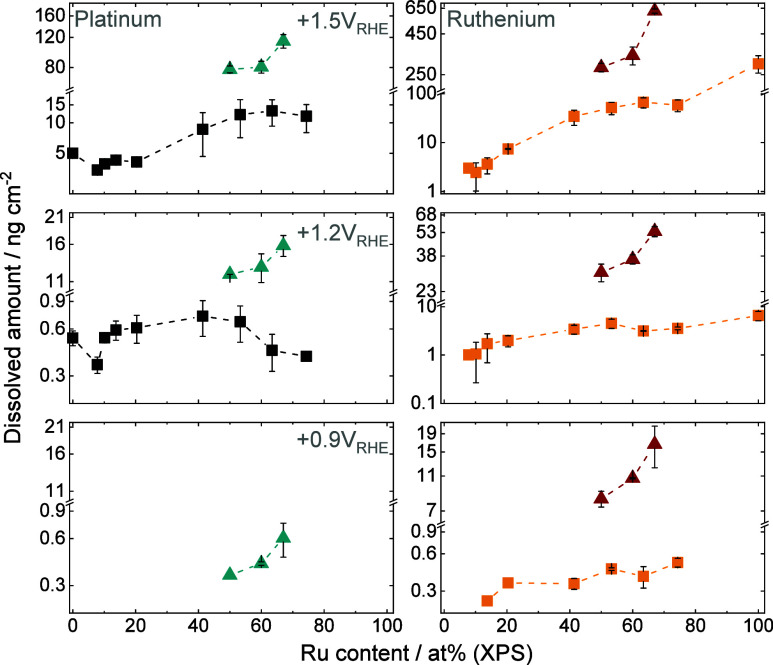
Amount of dissolved Pt (left side) and Ru (right side) during one cycle calculated from the dissolution curves presented in Figure 3. The amounts dissolved were obtained by integrating the dissolution data recorded over the whole cycle. Dark blue and yellow squares – sputtered thin-film samples; teal and red triangles – C-supported NPs. Error bars were calculated from at least two measurements, each performed on a fresh sample. Dashed lines connecting the points serve only as guides for the eye of the reader.

The dissolution data recorded have been analyzed only qualitatively so far. The dissolution curves are now integrated to support the qualitative conclusions made above. First, results calculated for the three CVs recorded by gradually increasing the UPL from +0.90 to +1.50 V_RHE_ are discussed (Figure 4). It is visible that no Pt dissolution was detected during the first CV (to +0.90 V_RHE_ UPL) in the case of the sputtered samples, regardless of the Pt content of the alloy. Pt dissolution was clearly identified for the C-supported NPs. The amount of dissolved Pt followed the amount of Pt in the alloy in reverse order. Pt dissolution was quantifiable for all sputtered samples containing Pt when we increased the UPL to +1.20 V_RHE_. When the alloy contained the least amount of Ru (10 at. %), the amount of dissolved Pt dropped. However, when the amount of Ru was further increased, the quantity of dissolved Pt became similar to that of the pristine Pt sample. Pt dissolution suddenly increases when the Pt:Ru ratio approaches one, exceeding the values calculated for the pristine sample. This increase lasts until 36 at. % Pt content. As mentioned in Section 3.3, Ru dissolution considerably influences the amount of dissolved Pt if Ru is in excess, which is reflected in the dissolved amount values. Similar trends can be identified when the UPL is shifted to +1.50 V_RHE_ with one exception: instead of decreasing, the amount of dissolved Pt stabilizes at the highest Ru contents. Pt dissolution is approximately 1 order of magnitude higher in each C-supported sample than that in the planar model system and increases in a similar fashion elaborated previously for the CV recorded between +0.05 V_RHE_ and +0.9 V_RHE_.

The picture is different for Ru dissolution (right panel in Figure 4), where ≈0.3–0.5 ng cm^–2^ Ru dissolution was determined even at the lowest UPL. Ru dissolution slowly increases (from 1 ng cm^–2^ to 6.5 ng cm^–2^) with the Ru content (linear relationship) if UPL was set to +1.20 V_RHE_. The highest dissolved amounts are always obtained for the pristine Ru sample. The amount of Ru dissolved scales exponentially with the Ru content when the UPL is increased to +1.50 V_RHE_, starting at 3 ng cm^–2^ (10 at. % Ru content) and reaching as much as 292 ng cm^–2^ (pristine Ru). This is at least 1 order of magnitude higher than the amount of dissolved Pt, so it is unsurprising that Ru dissolution significantly influences the extent of Pt dissolution. A similar increase is visible for the C-supported NPs, for all three CVs (especially in the case of the sample containing 63 at. % Ru).

As the next step, the dissolution rates were integrated for the AST step (all 30 cycles together). The dissolved amounts calculated are presented in Figure S8. Dissolved Pt amounts decrease in a similar fashion when a small amount of Ru is introduced in the crystal lattice, and this decrease lasts until the Ru content of the Pt_*x*_Ru_*y*_ alloy reaches 40 at. %. The amount of dissolved Pt exponentially increases from this point, reaching about 62 ng cm^–2^ at 74 at. % Ru content. 262 ng cm^–2^ Pt dissolves from the C-supported Pt_50_Ru_50_ alloy during the entire AST, which increases to 323 ng cm^–2^ in the case of the Pt_37_Ru_63_ sample. In contrast to this, Ru dissolution shows a continuous, steady increase, but when the Ru content of the alloy reaches 40 at. %, this increase levels down but is still notable (303 ng cm^–2^ Ru dissolved for the Pt_60_Ru_40_ sample vs 535 ng cm^–2^ calculated for pristine Ru). Ru dissolution is significantly higher when Ru content rises from 50 to 63 at. % in the case of the C-supported NPs. The dissolved Ru *doubles* by increasing the Ru content by only 13 at. %. Such differences were not experienced in the case of the sputtered thin films, warning us to approach the transferability of the results gathered for the model library to C-supported NPs. It indirectly suggests that the stability of Ru-rich systems can drastically drop by even a little bit of decrease in the Pt:Ru ratio.

Next, we evaluated the dissolution during CV after the AST step. The determined dissolved amounts were compared to those calculated for a similar CV (recorded applying a 10 mV s^–1^ scan rate between +0.05 V_RHE_ and +1.50 V_RHE_) prior to the AST step (Figure S9). Dissolved amounts of Pt pre- and post-AST are almost identical. A slight decrease occurs (between 20 and 25%) only when the Ru content exceeds 54 at. %. In contrast, Pt dissolution decreases by almost 60% (!) in the case of the C-supported NPs. This means that either the Pt leaching from the alloys is so extensive that there is a limited amount of Pt atoms accessible or that Pt remains in the alloy in a form that cannot be dissolved during the CV protocol. As opposed to Pt, the amount of dissolved Ru decreases notably in the case of the sputtered library (varying between 30 and 40%), regardless of the Ru content. The only sample that does not fit this trend is pristine Ru, where Ru dissolution decreases by 75% (from almost 300 ng cm^–2^ to around 70 ng cm^–2^). This is additional proof that alloying Ru, even with the smallest amount of Pt, can significantly enhance the global stability of Ru. The amount of dissolved Ru decreases by almost 90% when the Ru content is between 50 and 60 at. % for the C-supported NPs. Interestingly, this decrease is about 70% for the sample containing the highest amount of Ru. It is visible that Ru leaches from the alloy even to a greater extent than Pt, and we speculate that the alloy completely depletes in Ru, hence the considerably smaller dissolved amounts. We can draw a similar conclusion regarding the dissolution features. While dissolution peaks are clearly visible for both Pt and Ru before the AST step, these features are considerably smaller for Pt. They almost completely vanish for Ru during the CV recorded after it.

The electrochemical protocol also significantly affects the composition of the C-supported NPs, as evidenced by HAADF-STEM-EDX measurements (Table S1). For example, the composition of Pt_33_Ru_67_ shifts to Pt_70_Ru_30_ at the end of the electrochemical protocol. It has to be noted here that this is not a “global” change in composition – the surface of the catalyst is affected much more than the core of the NP, leading to the enrichment of Pt at the electrocatalyst surface. Besides the Pt:Ru ratio, the total mass percentage of metal lost was also calculated (Figure S10B). This value is negligibly small in the case of the Pt-rich samples (maximum 0.2 wt %), but it rapidly increases with the Ru content in the alloys, reaching more than 1% for the Pt_26_Ru_74_ sample. These values are even more striking for the C-supported NPs ranging from 5.5 to 9.6 wt % in the case of the Pt_50_Ru_50_ and Pt_33_Ru_67_ samples. These observations underline the conclusion that inappropriate operating conditions in real devices could easily lead to catalyst dissolution and, in the end, rapid device failure.

Our results can be summarized in the following points:

Higher Pt and Ru dissolution was observed for the C-supported Pt_*x*_Ru_*y*_ NPs compared to the sputtered thin film library.
Pt and Ru dissolution trends are completely different for the sputtered and C-supported samples.
Significantly more Pt and Ru leached out from the C-supported alloy samples compared to the sputtered ones as a result of the AST step. This was evidenced by comparing the dissolved amounts calculated for CVs recorded applying identical parameters before and after the AST step.

### 3.5 Changes in Morphology and Composition after the Electrochemical Protocol

**Figure 5:**
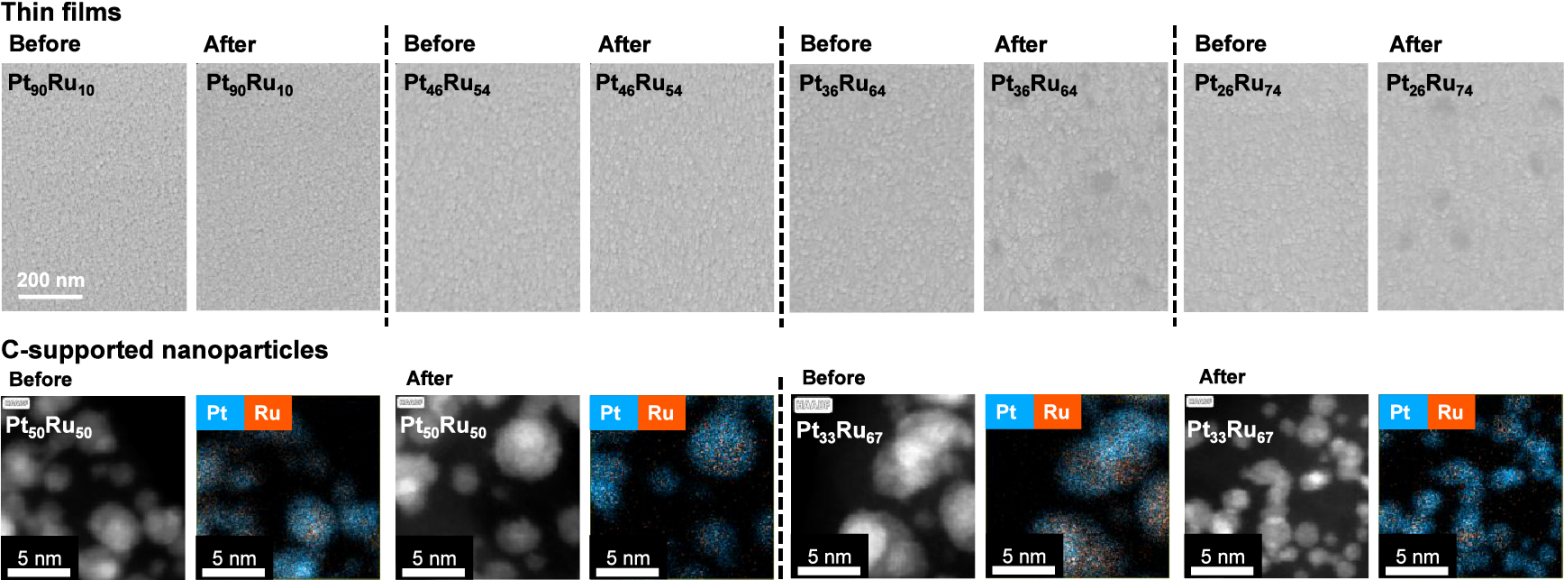
SEM images captured for the sputtered thin films (top row) before and after performing the electrochemical protocol presented in Figure 3. HAADF-STEM and PtRu spectral images recorded for the C-supported NPs (bottom row) before and after performing the electrochemical protocol presented in Figure 3.

All samples were analyzed further after the electrochemical protocol to determine whether the differences observed in dissolution characteristics for the sputtered thin films and the NPs are manifested in morphology and composition. The morphology of the sputtered thin films was scrutinized with SEM (Figures 5 (top row) and S2). No changes in the surface characteristics were observed in the case of the Pt/rich compositions. However, when the amount of Ru exceeded 50 at. %, several pits developed at the electrode surface, which is evident as dark spots with a diameter of at least 30–50 nm. The development of morphology parallels the alloy composition and is also manifested in the electrochemical behavior: while CVs recorded before and after the AST step are more or less identical for the Pt-rich samples, quite a few changes can be observed when the amount of Ru exceeded Pt (CVs are presented in Figure S11). The area of each post-AST CV is larger than that measured before the AST step, indicating an increase in the ECSA because of extensive leaching of Ru from the alloy structure. The only exception is pristine Ru, where the area of the measured CVs is similar. However, the onset of the OER (accompanied by Ru dissolution) shifted toward more positive potential values. It is also visible that as a result of the AST step, the oxide reduction peak position shifted toward more positive values for all samples. This means that the Pt:Ru ratio increased at the electrocatalyst surface.

This picture is different for C-supported PtRu NPs. According to the HAADF-STEM images presented in Figures 5 (bottom row), S12, and S13, Ru was evenly dissolved from the Pt_50_Ru_50_ NPs, decreasing the Pt:Ru ratio from 1:0.96 to 1:0.33. When the amount of Ru exceeded 50 at. %, the amount of dissolved Ru increased considerably. Ru leached from the alloy samples unevenly, forming a Pt-rich shell structure, as evidenced by the spectrum images. The trend regarding the electrochemical behavior contradicts the one observed for the sputtered thin films: in the case of the CVs recorded after the AST (Figure S11), current densities above +1.30 V_RHE_ decreased considerably along with the overall area of the voltammograms regardless of the Ru content of the alloys. The reason behind this is the severe decrease in the Ru content of the alloys (Pt:Ru ratio decreased from 1:0.96 to 1:0.33, from 1:1.44 to 1:0.4, and from 1:1.90 to 1:0.43 for the Pt_50_Ru_50_, Pt_33_Ru_66_, and Pt_25_Ru_75_ samples, respectively, see Table S1 for further details), since the high current densities above +1.23 V_RHE_ stem from both the OER progressing at a high rate and the extensive dissolution of Ru. As mentioned in the previous section, Ru is not stable above +1.40 V_RHE_ due to the formation of RuO_4_ species, leading to constant Ru dissolution (instead of a transient phenomenon common for Ru below this potential).^17,21^ Additionally, the oxide reduction peak shifts toward more positive potentials for all three alloys, signaling the enrichment of the alloys in Pt (see Figures 2 and S11).

Three different morphologies were identified for dealloyed PtCo and PtCu, which heavily depend on the size of the original alloy nanoparticles.^7,24,27,30,56^ A single core–shell structure was observed when the size of the nanoparticles was smaller than 10–15 nm. Irregular-shaped multiple core–shell NPs were found when the size of the NPs exceeded 15 nm. Finally, surface pits and nanopores were found when the particle size was considerably larger (15–30 nm and above). It has to be noted here that the amount of the less noble constituent (i.e., Co, Cu, and Ni) was always higher than that of Pt. Several similarities can be identified between the published findings on these systems and the PtRu samples investigated in our study. First, evidenced by STEM-EDXS measurements, a surface enriched in Pt structure was formed when the amount of Ru was higher than that of Pt. The three carbon-supported PtRu NP samples are intended to be used in direct alcohol fuel cells and electrolyzers utilized for electrosynthesis purposes. Thus, the average particle size of the alloy NPs is less than 10 nm. Hence, it is a range in which single core–shell NP formation was identified for M-rich PtM alloys (M = Ni, Co, Cu). The development of such structures cannot be observed for the Pt_50_Ru_50_ sample, indirectly indicating a notable alloy stabilization if the amount of the more stable element is equal to or greater than the less noble one. The evolution of the morphology is completely different for the sputtered thin films. Again, when the PtRu samples contained more than 50 at. % Pt, the morphology of the thin films remained identical to the ones recorded for the pristine samples prior to getting in contact with the electrolyte. However, when the amount of Pt decreases below 50 at. %, several deep surface pits formed (30–50 nm in average diameter). These are similar to what was observed for bigger PtM NPs (*d* −100 nm), in which case the inner sections of the NPs bear bulk-like properties. These surface pits appeared as cracks in the case of Ni- and Cu-rich PtNi and PtCu samples.^54,57,58^ Crack formation was always accompanied by an increase in the grain sizes. The size of the cracks and the development of the overall surface morphology highly depended on two aspects: the amount of the less noble component in the alloy and the applied electrochemical protocol (number of cycles and upper potential limit). The surface crack formation was first spotted when the amount of Ni in the Pt_*x*_Ni_*y*_ alloy exceeded 40 at. %.^57^ In contrast, the drastic metamorphosis of Pt_*x*_Ru_*y*_ started only when the Ru content of the alloy reached 60 at. %. While surface pits are unequivocally present, no grain size increase was evidenced in the case of the samples studied in this paper, regardless of the Ru content. It is probably due to the nature of the applied electrochemical protocol: the AST protocol presented in our study was significantly shorter compared to others (30 cycles vs several hundred or thousands of cycles). Since the sputtered samples are composed of a continuous PtRu layer with position-dependent composition (similar to a plate-like electrode made out of one piece of metal(alloy), for example), they can model well the behavior of larger bimetallic particles. All in all, based on our results, it seems that correlations found for the dealloying characteristics of noble–non-noble PtM NP systems can be applied to bimetallic noble metal alloys in which one of the alloy components is notably less stable than the other constituent.

### 3.6 Influence of Isopropanol on Stability

Several additional factors to electrochemical parameters can influence the stability of a given anode electrocatalyst, especially if we are talking about real devices and real operating conditions. One such factor that must be considered is the presence of substrate molecules and the intermediates and products formed during the electrocatalytic reaction. PtRu is a state-of-the-art electrocatalyst in terms of alcohol oxidation and is the typically applied anode catalyst in direct alcohol fuel cells. In our previous research projects,^14,20,59^ PtRu was utilized as an electrocatalyst to oxidize isopropanol to acetone selectively. An identical electrochemical protocol, as presented in Figure 3, was performed by adding 0.05 M isopropanol to the 0.1 M HClO_4_ electrolyte. Measurements were carried out using both the sputtered PtRu material library and C-supported NPs.

**Figure 6:**
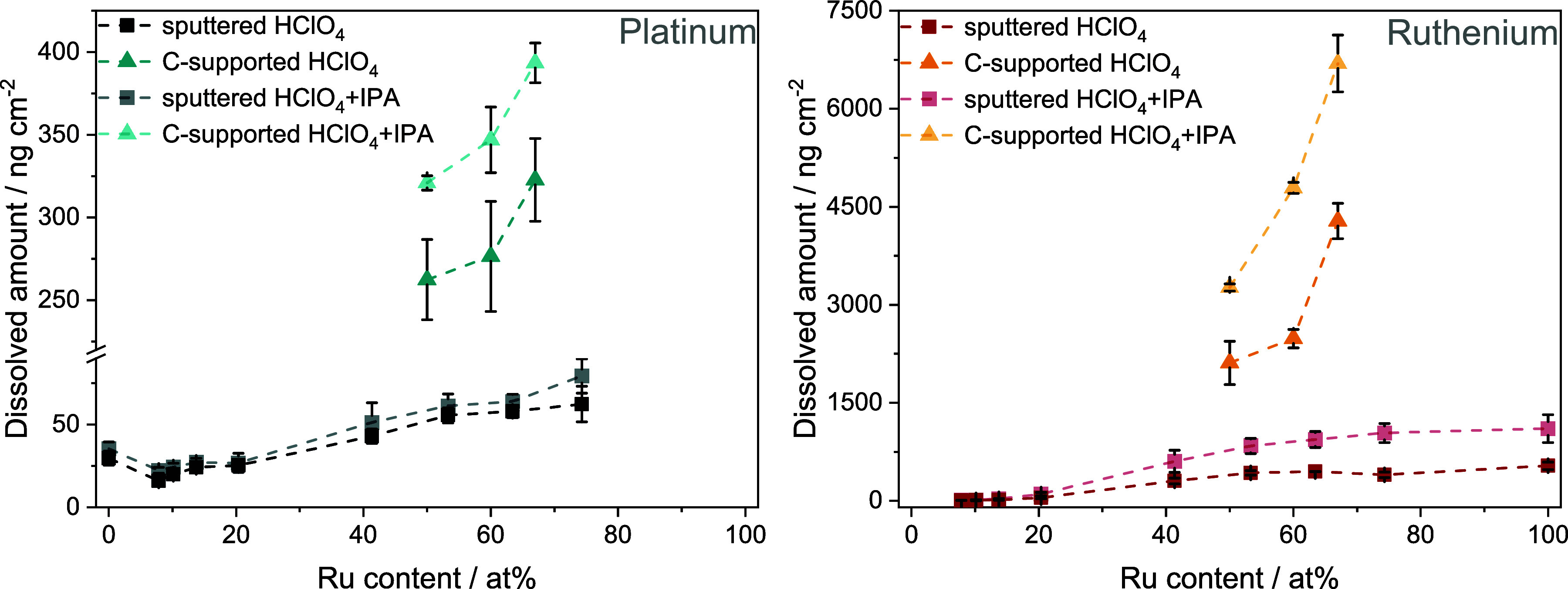
Dissolved amounts of Pt (left) and Ru (right side) during the whole AST step for the sputtered thin films and C-supported NPs calculated by integrating the dissolution rates presented in Figures S14 and S15. Error bars were calculated using data from at least two measurements, performed each using a fresh catalyst spot. Dark symbols – data recorded in 0.1 M HClO_4_, pale symbols – data recorded in 0.1 M HClO_4_ + 0.05 M isopropanol. Squares – sputtered thin films; triangles – C-supported NPs. Dashed lines connecting the points serve only as a guide to the eye of the reader.

The recorded dissolution rates are presented in Figures S14 and S15. As an example, the amount of dissolved Pt and Ru during the AST step, in both the presence and absence of 2-propanol, is shown in Figure 6. The amount of Pt only slightly increases in the presence of isopropanol, and this increase can be clearly spotted only when the amount of Pt is less than 50 at. % in the alloy. In contrast, a striking increase in the mass of dissolved Ru can be detected if the amount of Ru exceeds 40 at. % in the PtRu alloy, sometimes reaching even twice the values in the absence of isopropanol. Similar conclusions can be made for the carbon-supported NPs. It seems that isopropanol has a specific interaction with Ru atoms, most likely located at the surface of the alloy electrocatalyst, destabilizing them. A possible explanation could be that in the absence of isopropanol, oxygenated species are present at the surface of Ru under high anodic potentials, passivating it. These are consumed by the alcohol oxidation process, preventing the passivation, hence the protection of the Ru surface, leading to enhanced Ru dissolution. The exact mechanism of this process is yet unknown. However, increased Pt and Ru dissolution has also been observed in the presence of methanol (destabilization was tied to CO intermediate adsorption in that case).^45^ This phenomenon is currently under closer investigation in our lab. Our data demonstrates that the stability of the anode electrocatalyst can be significantly influenced just by the composition of the catalyst and the presence of isopropanol. Therefore, these factors must be considered when designing and selecting the optimal electrocatalyst for the given process.

## Conclusion and Outlook

The stability of a magnetron-sputtered Pt_*x*_Ru_*y*_ material library and three commercially available carbon-supported Pt_*x*_Ru_*y*_ NPs was studied in highly acidic electrolytes. We found that as a result of the electrochemical protocol, significantly more Pt and Ru dissolved from the C-supported samples compared to the sputtered ones. This was especially true for the AST steps. Additionally, dissolution trends were different for the thin films and the NPs. While the amount of dissolved Ru slowly increased with the Ru content in the sputtered bimetallic alloy films, even a small increase (e.g., 50 at. % to 60 at. %) in its amount caused a significant decrease in the stability of the alloy in the case of the NPs. The alteration in the Pt:Ru ratio was always accompanied by a mass loss. While this was equal to or below 0.1% for the Pt-rich samples, it rapidly increased with the increasing Ru content, reaching almost 2% for the pristine Ru sample. The mass lost because of the electrochemical protocol reached almost 10% (!) in the Pt_33_Ru_67_ C-supported NP sample case. The morphology and composition of the thin films and C-supported NPs were studied. The formation of a surface enriched in the Pt structure was observed for the Ru-rich NPs, while the formation of 30–50 nm diameter surface pits was captured for the Ru-rich sputtered thin films. Morphological changes were accompanied by a serious decrease in the Ru content. The development of morphology for both types of samples is in good agreement with what was found for M-rich bimetallic PtM alloys, where M is usually a less noble transition metal (e.g., Ni, Cu, Co). This allows for predicting the degradation pathways of fully noble bimetallic electrocatalysts for which one of the constituents is less stable than the other (besides PtRu, e.g., PdAu or PdRh).

Finally, the stability of the sputtered thin film library and the C-supported NPs was tested in the presence of isopropanol. While the stability of Pt was only slightly influenced by isopropanol, Ru dissolution was significantly higher, sometimes resulting in two times higher values compared to the absence of the alcohol. This rather alarming phenomenon means that the Ru content of the alloy can greatly destabilize it, inducing unforeseen challenges for real-life applications.

Based on our findings, we propose the following guidelines to consider before applying the Pt_*x*_Ru_*y*_ system in real devices. First and foremost, it is important to define a potential window in which the catalyst remains stable under the process conditions. The positive boundary of this is marked by the onset potential of dissolution determined for the least stable element in the alloy. In our case, Ru dissolution from the C-supported NPs starts at approximately +0.76 V_RHE_; however, as stressed in the previous sections, this value is (i) determined by the sensitivity of the ICP-MS and (ii) most likely depends on the time scale. To determine the exact value, long-term measurements are necessary to be performed. Since metal dissolution increases rapidly above the onset, the recommended positive potential boundary can be set at least 100 mV below this value to +0.65 V_RHE_. Since Pt_*x*_Ru_*y*_ is typically applied in electrocatalytic alcohol oxidation reactions, this criterion can be fulfilled under normal operating conditions in a DAFC. However, there are certain operation scenarios when the anode catalyst layer might experience considerably higher potentials, for example, at high loads, fuel starvation, and at the onset of the ORR and metal oxidation (mixed potential) during shutdown (presence of O_2_ in the anode compartment of the cell). The community’s knowledge about the exact potential values under these circumstances is limited at best, justifying the need for stability measurement in the widest potential window possible. The Ru content in the bulk is crucial and even more so at the alloy surface. We have shown that stability can very rapidly decrease even by a minor change of composition in favor of Ru and that even small amounts of uncertainties/impurities (e.g., phase separation or Ru accumulation at the catalyst surface) can lead to serious Ru dissolution and, in the end, faster electrocatalyst degradation than expected. Therefore, paying attention to the synthesis (and, as importantly, the post-synthesis handling) and characterization process (careful determination of surface composition) of the bimetallic Pt_*x*_Ru_*y*_ electrocatalyst samples is immensely important. Moreover, our data clearly indicate that activity and stability are linked and, therefore, should be investigated together, underlining the need for coupled electrochemical techniques. With the effective application of these, an optimal composition should be determined, providing the highest possible activity with acceptable stability. Trends for the sputtered material library cannot be fully transferred to NPs (i.e., systems that are closer to real application scenarios). Therefore, compositions proven to be promising in model measurements should be tested in real devices under process conditions. We demonstrated that the presence of isopropanol could greatly destabilize Pt_*x*_Ru_*y*_, especially the Ru-rich compositions, which is rather unpleasant for a catalyst that is typically applied in alcohol oxidation reactions. As stressed above, it is unknown whether this is a characteristic effect of isopropyl alcohol or if this issue is present when using other alcohols. Therefore, the stability of the system has to be checked in the presence of the given reactant, and the optimal composition should be, again, selected not just based on activity but also on the stability of the given electrocatalyst-fuel combination. This could even be a problem when the PtRu anode is coupled to a cathode at which processes resulting in various organic liquid products (e.g., acetate, ethanol, methanol, and more complex organic molecules as a result of various electrosynthesis processes) occur. These can often crossover the membrane to the anode side, opening the possibility of further destabilizing the anode catalyst, meaning that the effect of all possible products (that can be possibly identified in the anolyte stream) on the anode catalyst stability should be scrutinized carefully. In the future, designing electrocatalysts with advanced properties must rely on establishing such structure–property relationships and checking the viability of model system measurements using the catalyst samples that are intended to be used in a real device mimicking real operating conditions.

## Supporting Information Available

Figures S1–S15 and Table S1, additional material characterization data: SEM-EDX measurements, SEM and HAADF-STEM images, cyclic voltammograms recorded for the sputtered thin-films, additional online ICP-MS data recorded for both the sputtered and carbon-supported NP thin films, dissolved amounts calculated for the electrochemical protocols, development of catalyst composition and total mass loss calculations, online ICP-MS data recorded in the presence of isopropanol (PDF)
