# Supplementary material for: Stability of Bimetallic PtxRuy – From Model Surfaces to Nanoparticulate Electrocatalysts
Source: ACS Mater Au. 2024 Jan 16;4(3):286–99. doi: 10.1021/acsmaterialsau.3c00092 (PMC11083114; doi:10.1021/acsmaterialsau.3c00092)
Supplement: Supplementary file 1 — mg3c00092_si_001.pdf [file mg3c00092_si_001.pdf]

SUPPORTING INFORMATION

Stability of Bimetallic Pt_xRu_y – from Model Surfaces to Nanoparticulate Electrocatalysts

Attila Kormányos,[1,2,] Pascal Büttner,[3] Michael Bosch,[3] Maria Minichova,[1,4] Andreas Körner,[1,4] Ken J. Jenewein,[1,4] Andreas Hutzler,[1] Karl J. J. Mayrhofer[1,4], Julien Bachmann,[3] and Serhiy Cherevko[1,*]*

[1]Helmholtz-Institute Erlangen-Nürnberg for Renewable Energy (IEK-11), Forschungszentrum Jülich, Cauerstr. 1, Erlangen, 91058, Germany

[2] University of Szeged, Department of Physical Chemistry and Materials Science, Aradi sq. 1, Szeged, 6720, Hungary

[3] Chemistry of Thin Film Materials, IZNF, Friedrich-Alexander-Universität Erlangen-Nürnberg (FAU), Cauerstr. 3., Erlangen, 91058, Germany

[4] Department of Chemical and Biological Engineering, Friedrich-Alexander-Universität Erlangen-Nürnberg, Egerlandstr. 3, Erlangen, 91058, Germany

*attila.kormanyos@szte.hu; *s.cherevko@fz-juelich.de

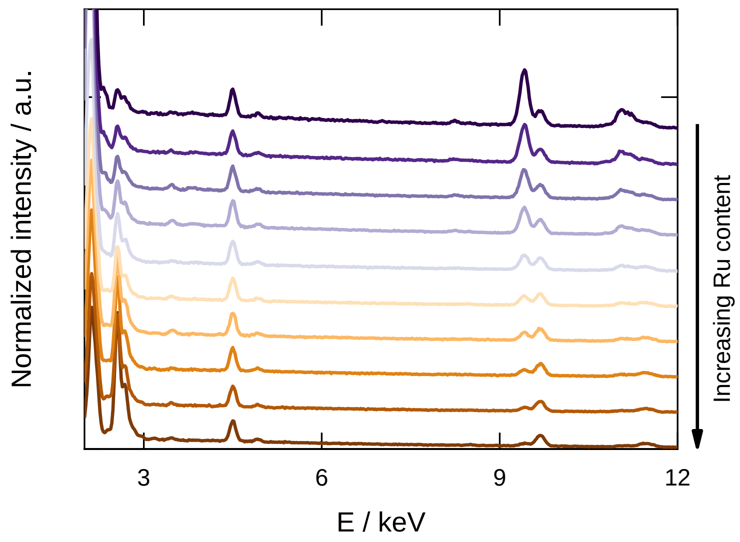

Figure S1. EDX spectra recorded for the sputtered thin film library.

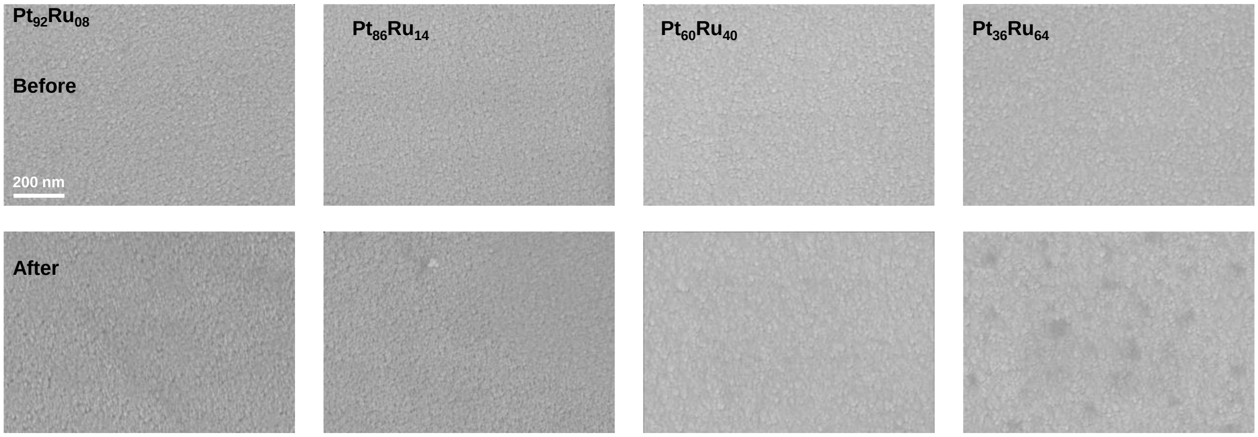

Figure S2. SEM images captured from the sputtered Pt_xRu_y material library before (top row) and after (bottom row) performing electrochemical protocols. Alloy compositions presented in each image are derived from the XPS data presented in Figure 1.

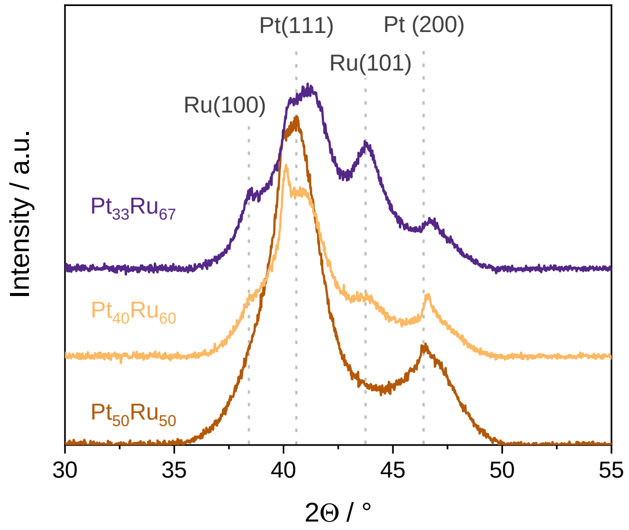

Figure S3. XRD data collected for the carbon supported Pt_xRu_y nanoparticles.

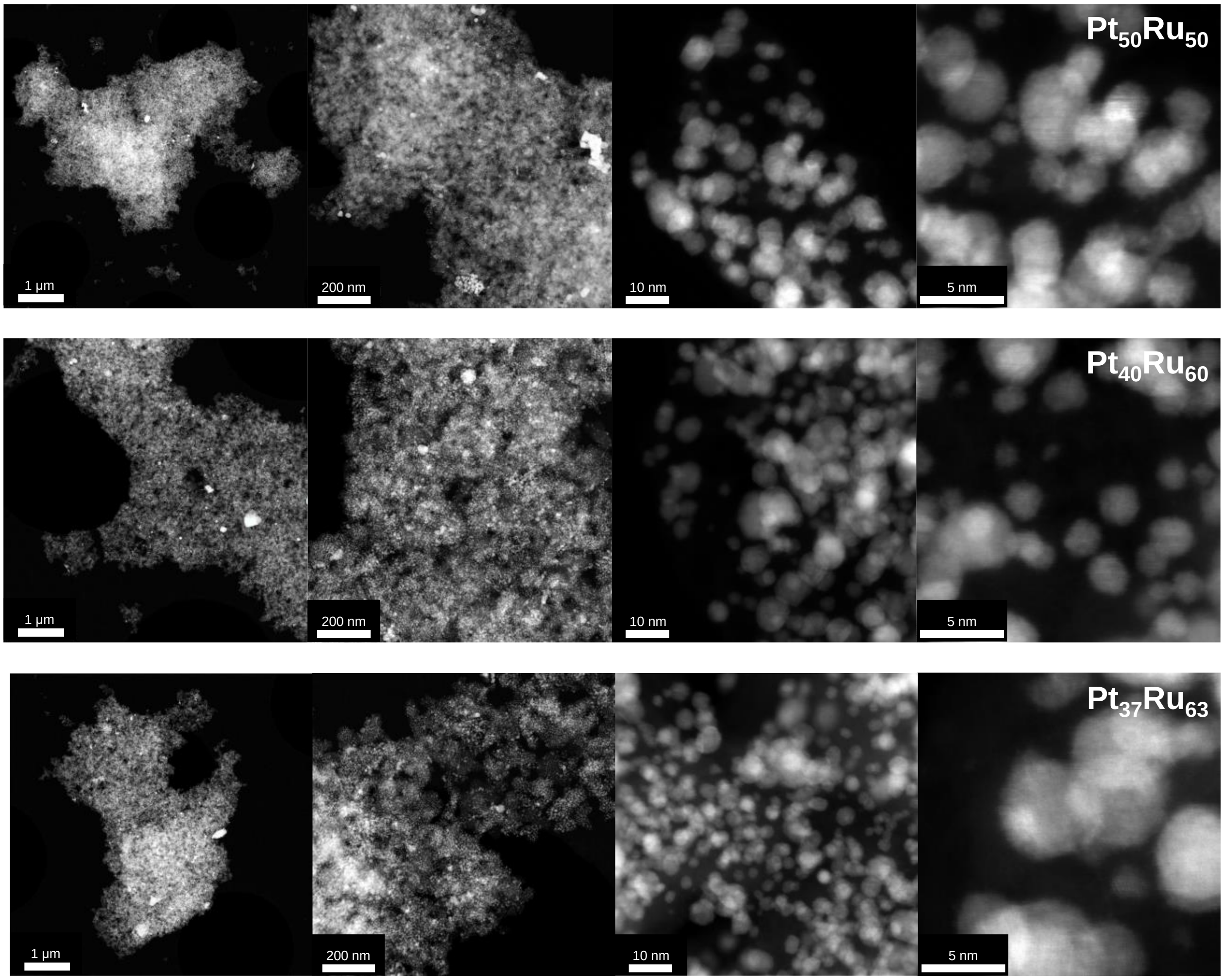

Figure S4. HAADF-STEM images captured from the C-supported Pt_xRu_y NPs.

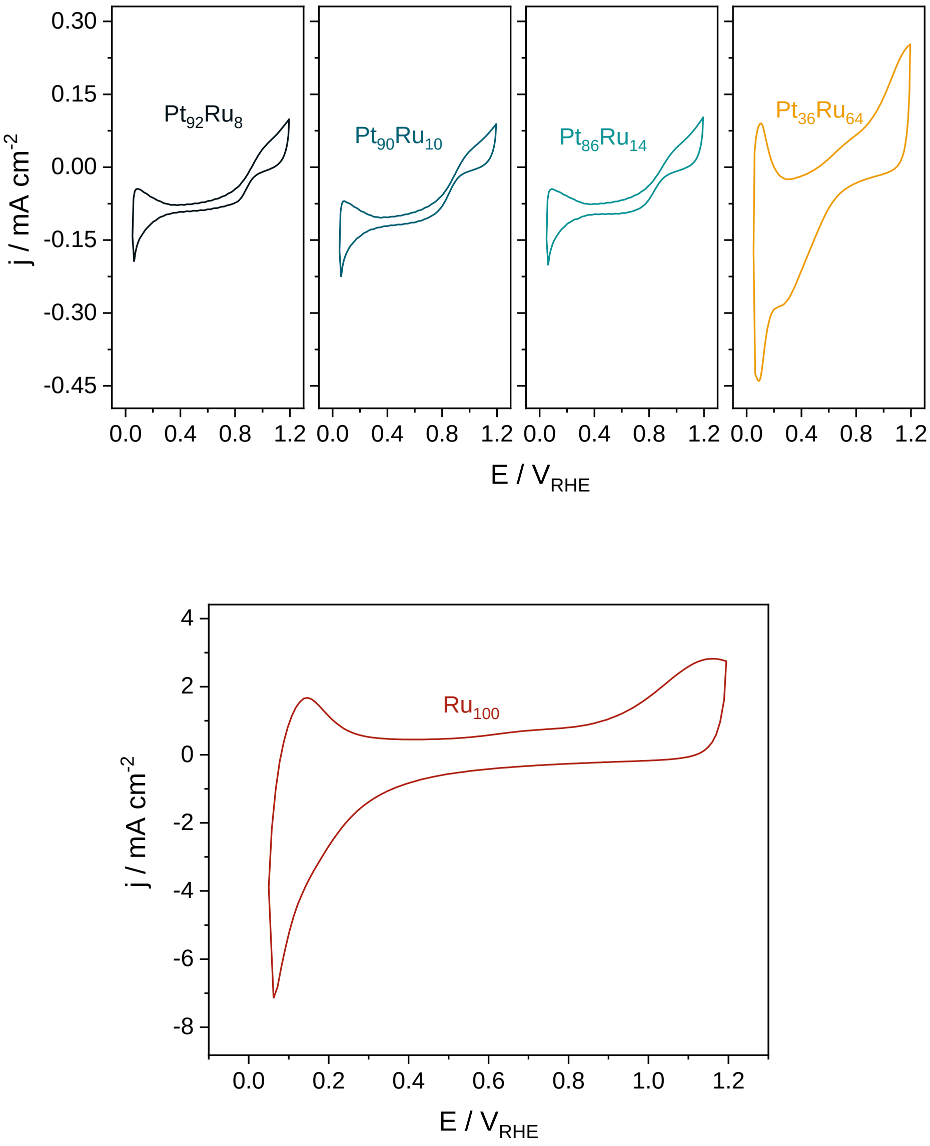

Figure S5. Cyclic voltammograms recorded for the sputtered thin films. CVs were recorded between +0.05 V_{RHE} and +1.20 V_{RHE} applying 50 mV s^{-1} scan rate in Ar-saturated 0.1 M $HClO_4$ electrolyte. Prior to recording the CVs presented here, a cleaning cycle was applied in between +0.05 V_{RHE} and +1.50 V_{RHE} applying 200 mV s^{-1} scan rate.

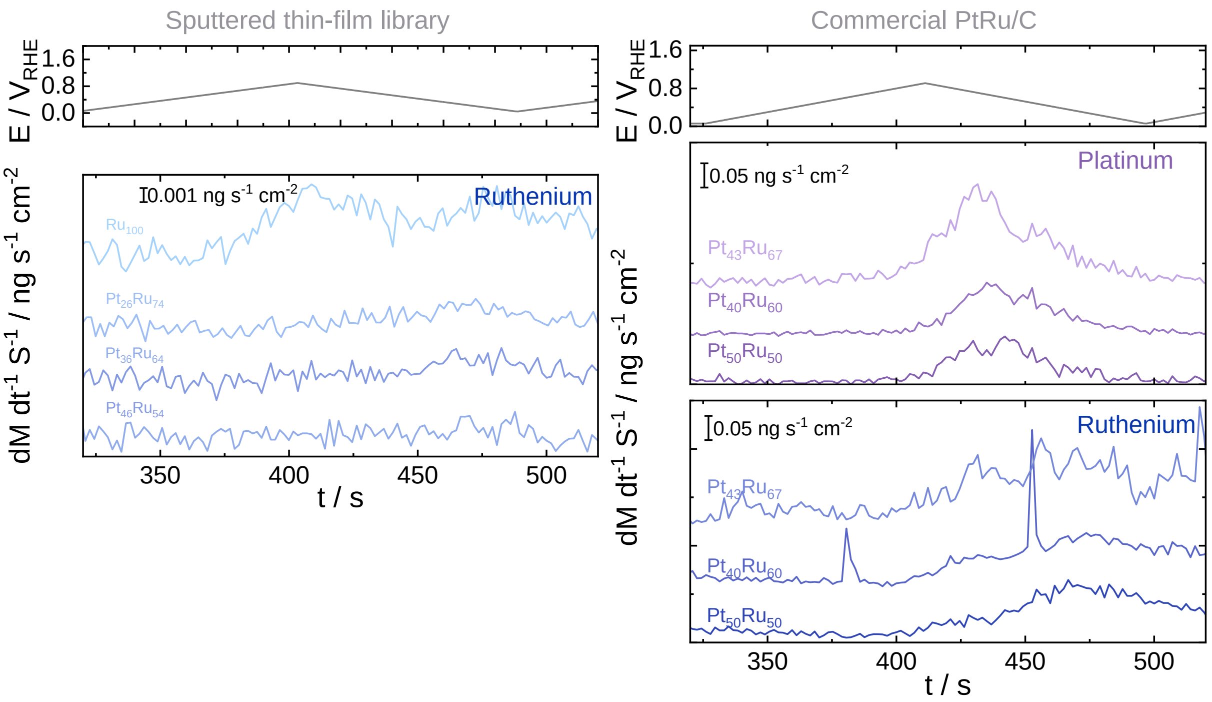
Sputtered thin-film library
Commercial PtRu/C
E / V_RHE
1.6
0.8
0.0
dM dt^-1 S^-1 / ng s^-1 cm^-2
0.001 ng s^-1 cm^-2
Ruthenium
Ru100
Pt26Ru74
Pt36Ru64
Pt46Ru54
0.05 ng s^-1 cm^-2
Platinum
Pt43Ru67
Pt40Ru60
Pt50Ru50
350
400
450
500
t / s

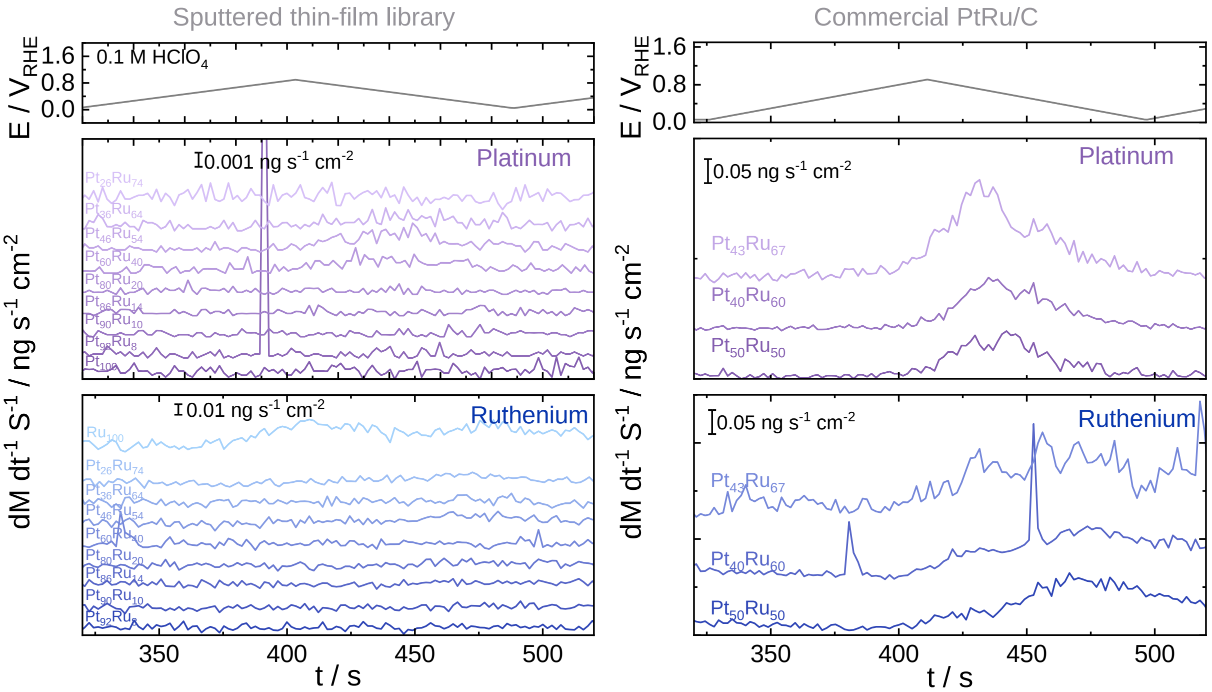
Sputtered thin-film library
Commercial PtRu/C
E / V_RHE
1.6
0.8
0.0
0.1 M HClO4
dM dt^-1 S^-1 / ng s^-1 cm^-2
0.001 ng s^-1 cm^-2
Platinum
0.01 ng s^-1 cm^-2
Ruthenium
Ru100
Pt26Ru74
Pt36Ru64
Pt46Ru54
Pt60Ru40
Pt80Ru20
Pt86Ru14
Pt90Ru10
Pt92Ru8
Pt100
0.05 ng s^-1 cm^-2
Pt43Ru67
Pt40Ru60
Pt50Ru50
350
400
450
500
t / s

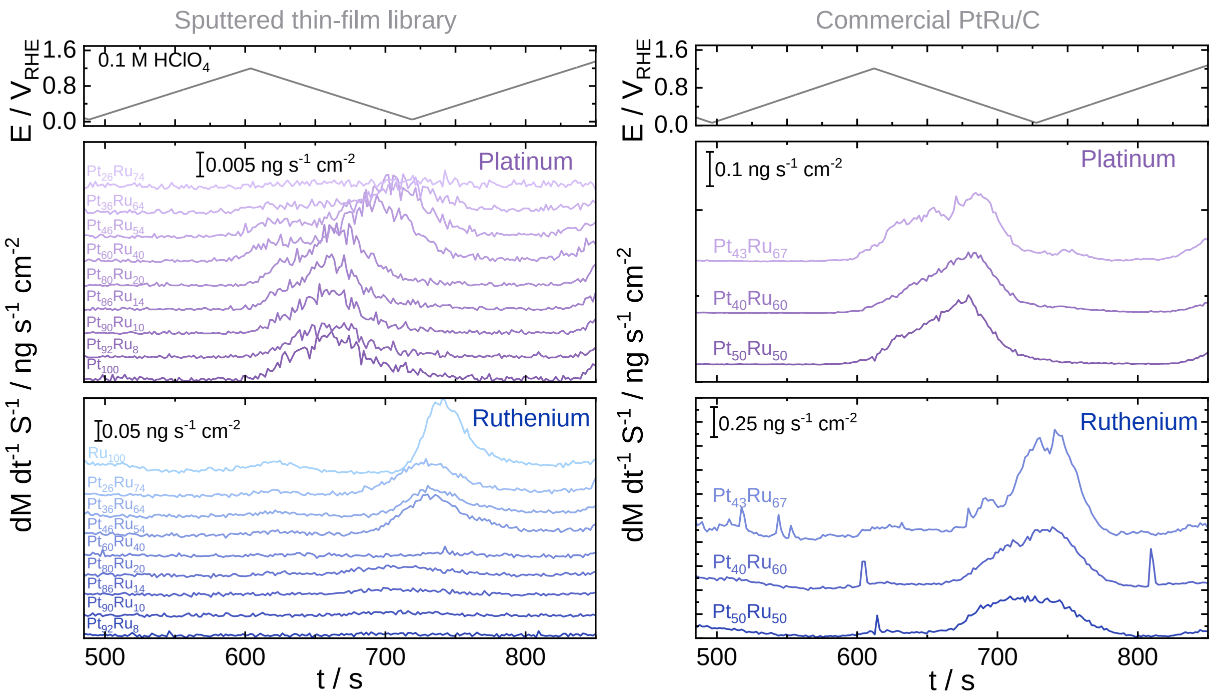

Figure S6. Magnified sections of Figure 3 presented in the main text.

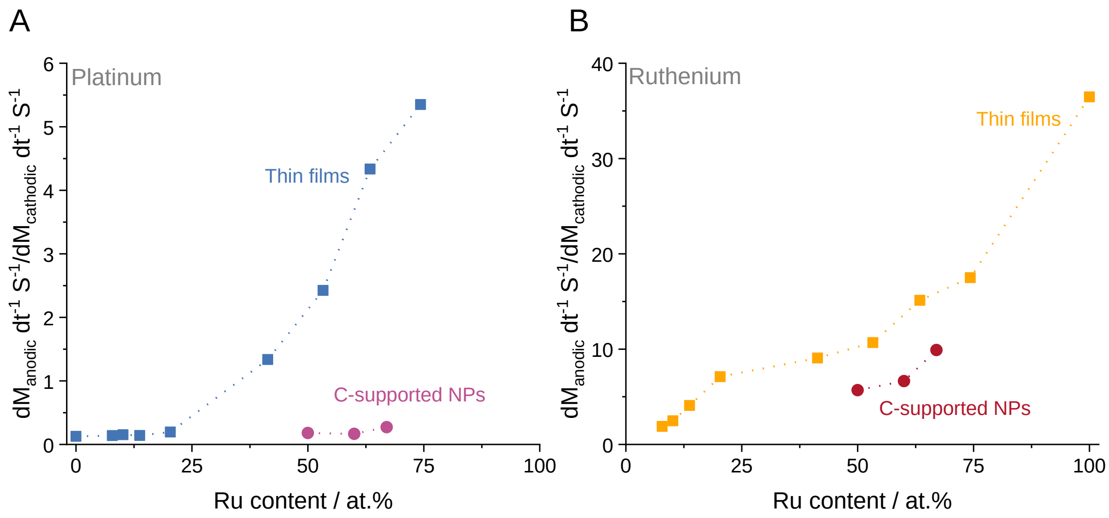

Figure S7. Platinum (A) and ruthenium (B) peak dissolution rate ratios (area of anodic dissolution peak divided by the area of the cathodic dissolution peak) calculated from the dissolution curves presented in Figure 3. All calculations were made using the third cyclic voltammogram recorded to E = +1.50 V_{RHE} upper potential limit applying 10 mV s^{-1} scan rate. Squares – data obtained for the sputtered thin films, Circles – data obtained for the C-supported NPs. Dashed lines connecting each point serve only as guides for the eye of the Reader.

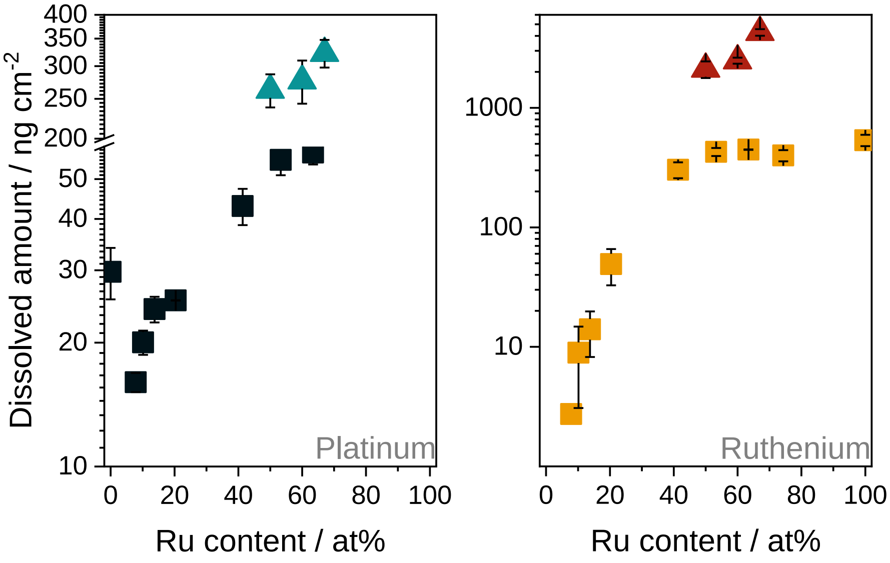

Figure S8. The amount of dissolved Pt (left side) and Ru (right side) during the AST step calculated from the data presented in Figure 3. Dissolved amounts were obtained by integrating the dissolution data recorded over the whole cycle. Dark blue and yellow squares – sputtered thin-film samples, teal and red triangles – C-supported NPs. Error bars were calculated from at least two measurements, always performed on a fresh sample. Dashed lines connecting each point serve only as guides for the eye of the Reader.

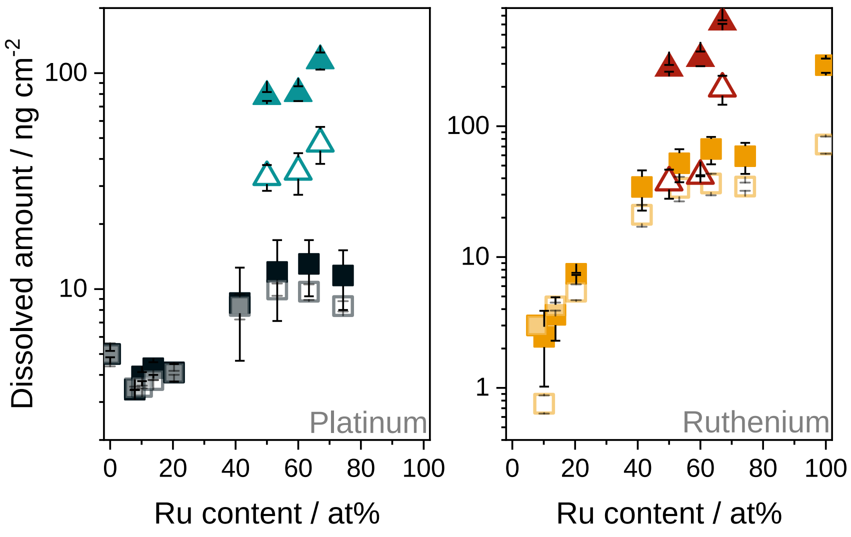

Figure S9. The amount of dissolved Pt (left side) and Ru (right side) during one cycle (recorded between +0.05 V_{RHE} and +1.50 V_{RHE} applying 10 mV s^{-1} scan rate) before and after the AST step calculated from the dissolution curves presented in Figure 3. Dissolved amounts were obtained by integrating the dissolution data recorded over the whole cycle. Dark blue and yellow squares – sputtered thin-film samples BEFORE the AST step, grey and light yellow squares – sputtered thin-film samples AFTER the AST step, teal and red triangles – C-supported NPs BEFORE the AST step, hollow teal and hollow red triangles – C-supported NPs AFTER the AST step. Error bars were calculated from at least two measurements, always performed on a fresh sample.

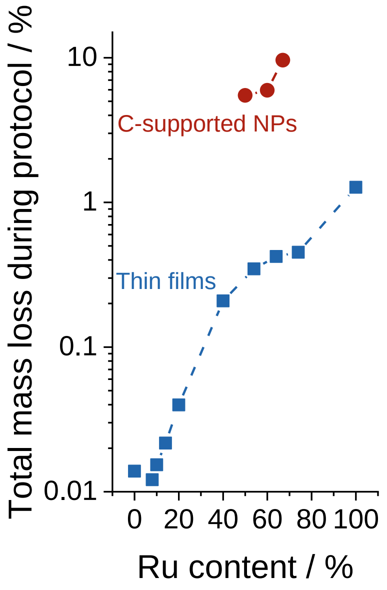

Figure S10. Total loss of metal from the given alloy during the electrochemical protocol.

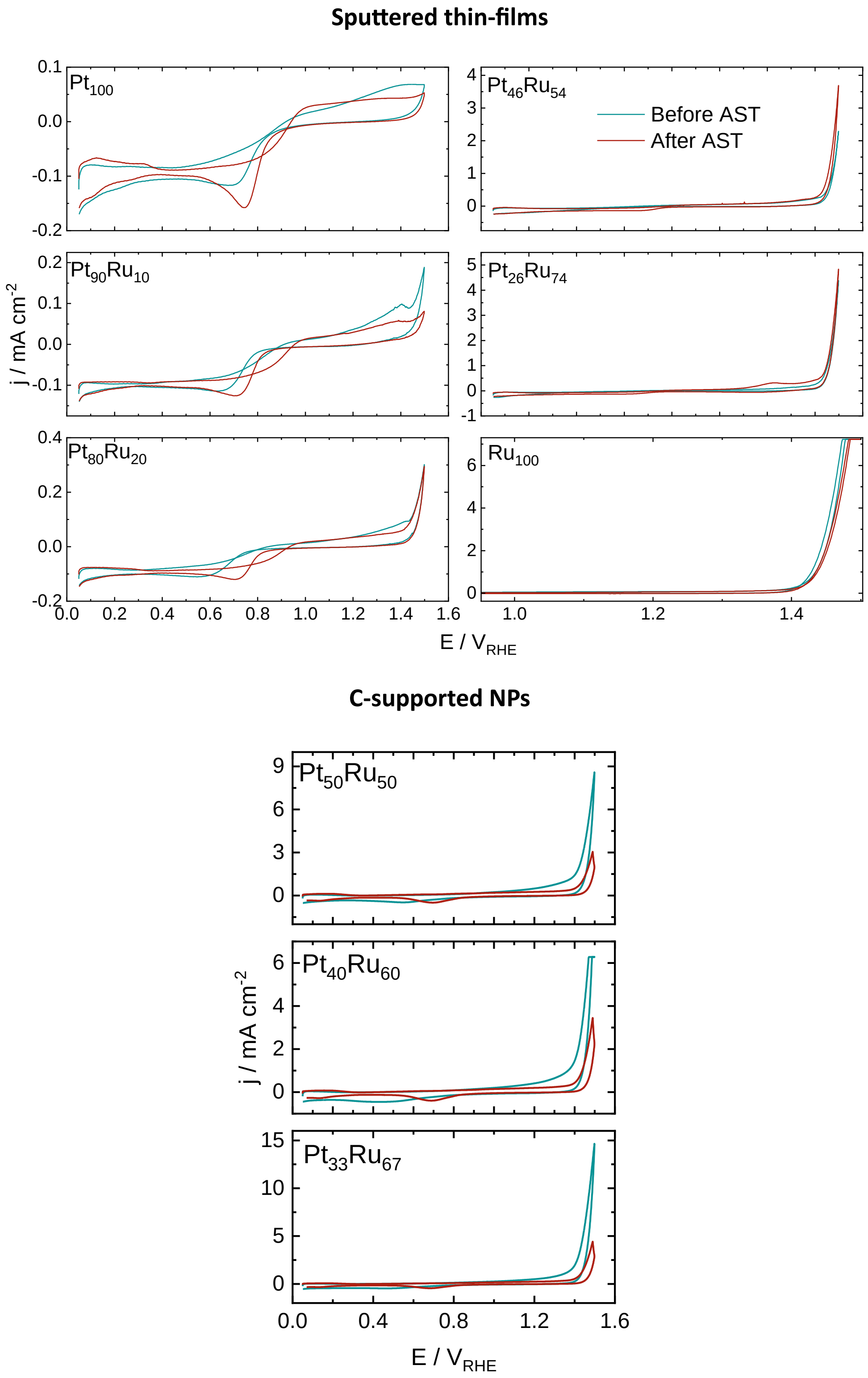

Figure S11. CVs recorded for the sputtered thin films (top panels) and for the C-supported NPs (bottom panel) before and after the AST step in between +0.05 V_{RHE} – 1.50 V_{RHE} applying 10 mVs^{-1} scan rate.

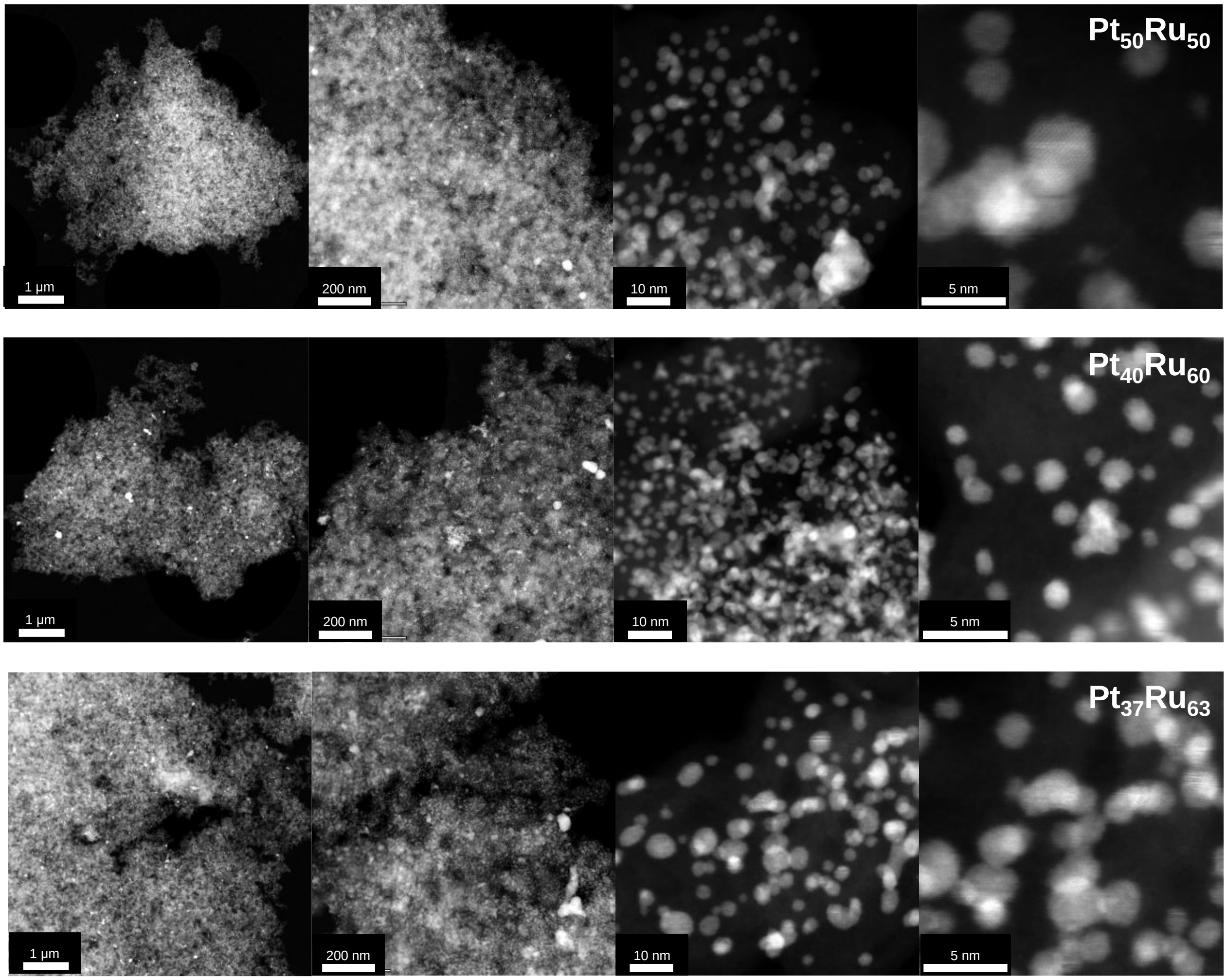

Figure S12. HAADF-STEM images captured from the C-supported Pt_xRu_y NPs after performing the electrochemical protocol.

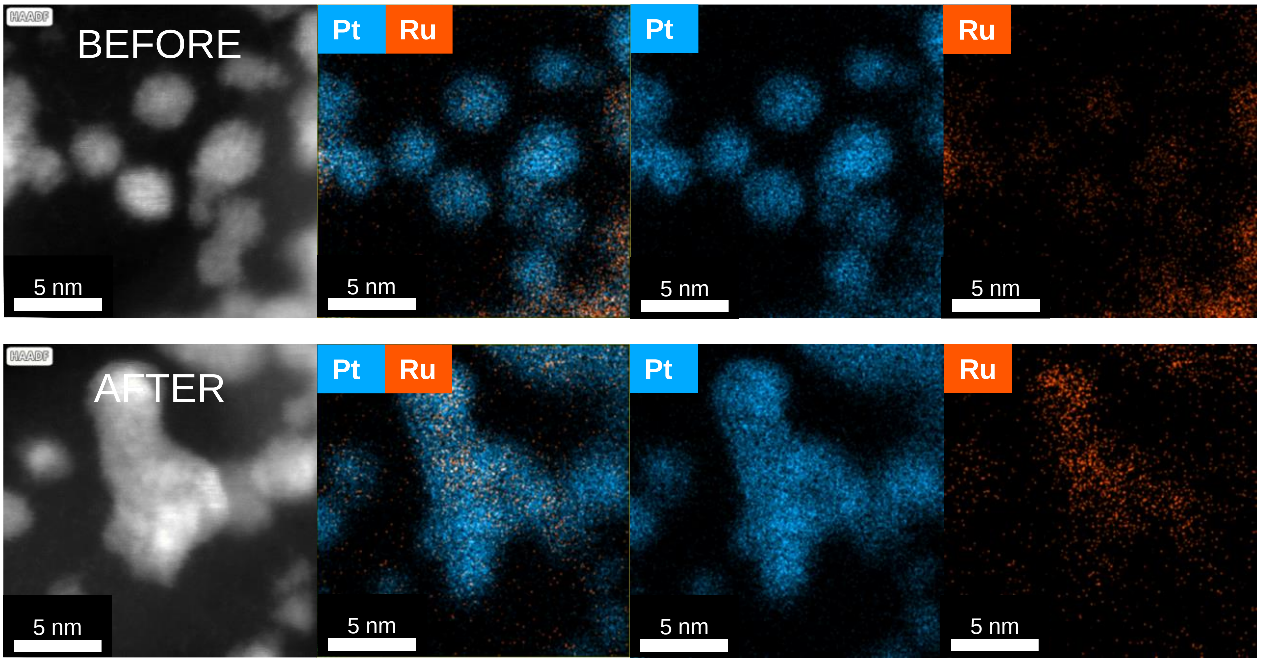

Figure S13. HAADF-STEM and PtRu spectral images recorded for the C-suported $Pt_{40}Ru_{60}$ sample before and after performing the electrochemical protocol presented in Figure 3.

Sample	Pt:Ru before the protocol	Pt:Ru after the electrochemical protocol
$Pt_{50}Ru_{50}$/C (Tanaka)	1:0.96	1:0.37
$Pt_{40}Ru_{60}$/C (Tanaka)	1:1.44	1:0.40
$Pt_{37}Ru_{63}$/C (Tanaka)	1:1.90	1:0.43

Table S1. Composition of the C-supported Pt_xRu_y NPs before and after performing the electrochemical protocol screened with STEM-EDXS.

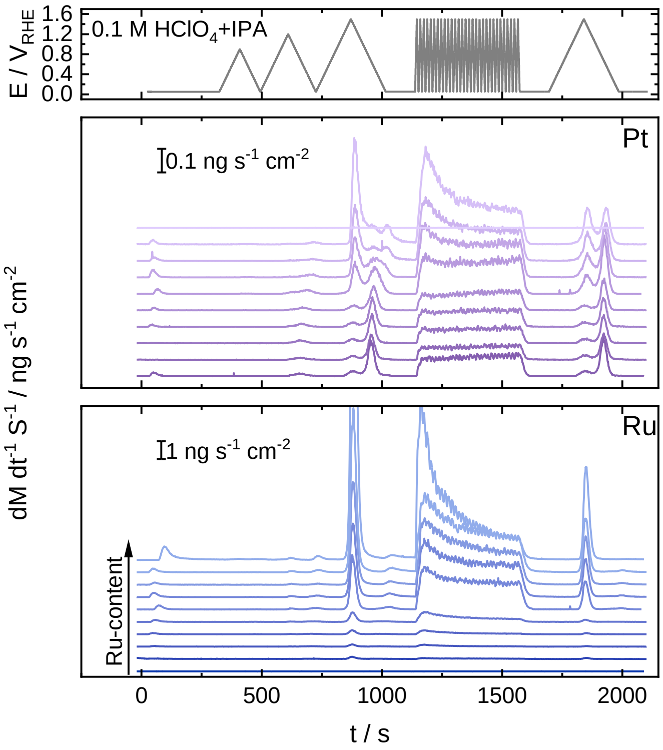

Figure S14. Top panel: Dissolution rates of Pt and Ru for sputtered Pt_xRu_y samples recorded during an electrochemical protocol consisting of three CVs with increasing upper potential limit from +0.90 V_{RHE} to +1.50 V_{RHE} (ΔE = 0.30 V, υ = 10 mV s^{-1}), and AST step where 30 CVs were recorded in between +0.05 V_{RHE} and +1.50 V_{RHE} applying 200 mV s^{-1} scan rate. The protocol was finished with a CV recorded in between +0.05 V_{RHE} and 1.50 V_{RHE} applying 10 mV s^{-1} scan rate. 0.1 M $HClO_4$+0.05 M isopropanol saturated with Ar was used as an electrolyte for all measurements.

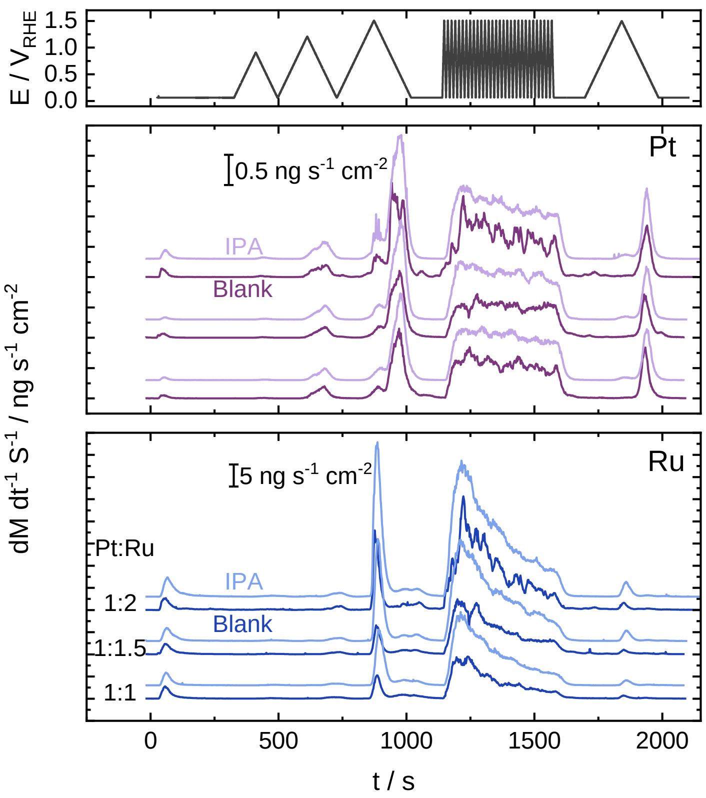

Figure S15. Top panel: Dissolution rates of Pt and Ru for C-supported Pt_xRu_y NPs samples recorded during an electrochemical protocol consisting of three CVs with increasing upper potential limit from +0.90 V_{RHE} to +1.50 V_{RHE} (ΔE = 0.30 V, υ = 10 mV s^{-1}), and AST step where 30 CVs were recorded in between +0.05 V_{RHE} and 1.50 V_{RHE} applying 200 mV s^{-1} scan rate. The protocol was finished with a CV recorded in between +0.05 V_{RHE} and +1.50 V_{RHE} applying 10 mV s^{-1} scan rate. 0.1 M $HClO_4$ and 0.1 M $HClO_4$+0.05 M isopropanol saturated with Ar was used as electrolytes for all measurements. Pt:Ru ratio is always presented on the left side of the graph.
